# An Epidemiological Model of Malaria Accounting for Asymptomatic Carriers

**DOI:** 10.1007/s11538-020-00717-y

**Published:** 2020-03-14

**Authors:** Jacob B. Aguilar, Juan B. Gutierrez

**Affiliations:** 1 Department of Mathematics and Sciences, Saint Leo University, Saint Leo, FL 33574 USA; 2 Department of Mathematics, University of Texas at San Antonio, San Antonio, TX 78249 USA

**Keywords:** Asymptomatic malaria, Relapse malaria, Naturally acquired immunity, Reproductive threshold, Sensitivity analysis, 37N25, 92D25, 92D30

## Abstract

Asymptomatic individuals in the context of malarial disease are subjects who carry a parasite load, but do not show clinical symptoms. A correct understanding of the influence of asymptomatic individuals on transmission dynamics will provide a comprehensive description of the complex interplay between the definitive host (female *Anopheles* mosquito), intermediate host (human), and agent (*Plasmodium* parasite). The goal of this article is to conduct a rigorous mathematical analysis of a new compartmentalized malaria model accounting for asymptomatic human hosts for the purpose of calculating the basic reproductive number ($\mathcal {R}_0$) and determining the bifurcations that might occur at the onset of disease-free equilibrium. A point of departure of this model from others appearing in the literature is that the asymptomatic compartment is decomposed into two mutually disjoint sub-compartments by making use of the naturally acquired immunity of the population under consideration. After deriving the model, a qualitative analysis is carried out to classify the stability of the equilibria of the system. Our results show that the dynamical system is locally asymptotically stable provided that $\mathcal {R}_0<1$. However, this stability is not global, owning to the occurrence of a sub-critical bifurcation in which additional non-trivial sub-threshold equilibrium solutions appear in response to a specified parameter being perturbed. To ensure that the model does not undergo a backward bifurcation, we demand an auxiliary parameter denoted $\varLambda <1$ in addition to the threshold constraint $\mathcal {R}_0<1$. The authors hope that this qualitative analysis will fill in the gaps of what is currently known about asymptomatic malaria and aid in designing strategies that assist the further development of malaria control and eradication efforts.

**Electronic supplementary material:**

The online version of this article (10.1007/s11538-020-00717-y) contains supplementary material, which is available to authorized users.

## Introduction

Malaria is one of the most lethal and complex parasitic diseases in the world (Gutierrez et al. 2015; World Health Organization 2019). Throughout human history, malaria has burdened most regions of our planet and has had a profound impact on human history and evolution; for example, it has been credited for contributing to the decline of the Roman Empire (Sallares 2002, p. 14). In 2018, the World Health Organization (WHO) reported the occurrence of approximately 228 million new cases of malaria (range 206–258 million), which resulted in an estimated 405 thousand disease-induced deaths (World Health Organization 2019). It was estimated that 67% of these fatalities were experienced by children under the age of five.

The life cycle of the *Plasmodium* parasite can be broken down into two separate sub-cycles: the asexual cycle, occurring in humans (intermediate host) and the sexual cycle in mosquitoes (definitive host), in which maturity is reached. The **sexual cycle** begins when a susceptible mosquito feeds on the blood of an infectious human, ingesting sexual forms of the *Plasmodium* parasite previously developed in the human body, known as *gametocytes*. While in the midgut lumen of the mosquito, these *gametocytes* fuse to form diploid *zygotes*, which grow into elongated *ookinetes*. The motile *ookinetes* burrow into the outer membrane of the mosquito midgut and form ellipsoid shaped *oocysts*. Eventually, the *oocysts* rupture releasing thousands of haploid forms called *sporozoites* (Rosenberg and Rungsiwongse 1991). These *sporozoites* accumulate in the salivary glands of the mosquito, causing it to become infectious.

The **asexual cycle** begins when an infectious mosquito bites the host and injects saliva with anticoagulant agents that keep the wound open, thus allowing a blood meal and simultaneously injecting *sporozoites* into the skin (Cowman et al. 2012). The *sporozoites* travel through the blood vascular system to the liver where they invade the cells of the liver, known as *hepatocytes*. Inside the human, a *Plasmodium* infection goes through two cycles: a initial liver (hepatic, or exo-erythrocytic) stage lasting a few days, followed by a blood (erythrocytic) stage that lasts until the host clears naturally the infections, receives treatment, or dies.

The **hepatic stage** begins in the *hepatocytes*, where a proportion of the *sporozoites* undergo a process called *pre-erythrocytic or hepatic schizogony*, in which they multiply asexually to produce thousands of haploid daughter cells, known as *merozoites*. During this process, *schizonts* are formed, causing the *hepatocytes* to rupture. This allows the *merozoites* to enter the bloodstream.

The **erythrocytic stage** begins when free-floating *merozoites* invade *erythrocytes* in a matter of minutes. Inside the *erythrocyte*, parasites enter the ring stage in which some mutate into an enlarged ring-shaped form called *trophozoites* that mature into *schizonts*, causing the cell to burst and releasing more *merozoites* into the bloodstream. In the case of *P. falciparum*, this process of invasion and rupture of RBCs occurs synchronously every 48 h (Mideo et al. 2013). At this point in the sub-cycle, a small portion of the *merozoites*, for reasons incompletely understood, develop into *gametocytes*, or sexual forms; however, no sexual reproduction occurs inside the host (John et al. 2006; Yan et al. 2015). The infected human host is now ready to infect new susceptible mosquitoes, thus completing the *Plasmodium* life cycle.

The blood stage parasites are responsible for most clinical symptoms associated with the disease (Arévalo-Herrera et al. 2015; Yazdani et al. 2006). The periodic rupturing of the RBCs results in the release of various debri and waste products, which in turn activate the immune system and cause symptoms such as chills, fatigue, pain, and fever. The average duration for the infection of an RBC is dependent on the *Plasmodium* species. *P. falciparum* has the interesting pathological effect of sequestration, which occurs when infected RBCs containing mature forms of the parasite, i.e., *trophozoites* and *schizonts*, adhere to the walls of small diameter blood vessels, e.g., the endothelium of capillaries and venules (David et al. 1983). As a result of sequestration, the microcirculation is reduced and in some cases inflammatory processes take place. One of the common complications of this sequestration is cerebral malaria, which might cause patients to sustain brain injury, resulting in long-term neuro-cognitive impairment (MacPherson et al. 1985).

A human host is called *asymptomatic* when it is a carrier for the *Plasmodium* parasite, but displays no clinical symptoms. Asymptomatic carriers contribute to *gametocyte* circulation by providing a hidden reservoir for the parasite to take refuge. As pointed out by Laishram et al. (2012b), asymptomatic infections often go undetected, resulting in a major source of *gametocytes* for local mosquito vectors. Accordingly, asymptomatic carriers contribute to the persistence of malaria transmission within their localized populations (Bousema et al. 2004). Frequent exposure to the *Plasmodium* parasites leads to naturally acquired immunity to the symptoms of the disease, but not necessarily to the parasite, and as a result, it creates asymptomatic carriers in a given population (Staalsoe and Hviid 1998).

Asymptomatic malaria infections have been reported in various high and intermediate transmission areas such as Kenya and Nigeria (Bousema et al. 2004; Eke et al. 2006). Recently, asymptomatic infections have been reported in relatively low endemic areas such as Colombia, Ecuador, and the Amazonian region of Brazil (Cucunubá et al. 2008; Coura et al. 2006; Sáenz et al. 2017; Lopez-Perez et al. 2015). There is much evidence that asymptomatic malaria infections play a fundamental role in malaria transmission (Lindblade et al. 2013). Disease transmission dynamics may be affected by the amount of asymptomatic carriers in a given population over a specified time interval. Indeed, a positive correlation between high transmission and high asymptomatic prevalence has been reported in Nigeria, Senegal, Gabon, and the Amazonian regions of Brazil (Alves et al. 2002; de Andrade et al. 1995; Dal-Bianco et al. 2007; Eke et al. 2006).

In accordance with Bruce-Chwatt et al. (1980), we define *malaria immunity* as the state of resistance to the infection brought about by all processes which are involved in destroying the *Plasmodia* or limiting their multiplication. *Natural innate immunity* is an intrinsic property of the host. This type of immunity is characterized by an immediate inhibitory response to the introduction of the parasite which is independent of any previous infection. Their are two types of acquired immunity, namely *active acquired immunity* and *passive acquired immunity*. *Active acquired immunity* is defined as an enhancement of the hosts defense mechanism due to previous contact with the pathogen. *Passive acquired immunity* is characterized by either the mother to child transfer of protective antibodies in the pre- or post-natal developmental periods or by the injection of such antibodies. In this work, we are focused on active acquired immunity, more specifically a type of immunity that is acquired through means of exposure.

Humans experience various kinds of *active acquired immunity* which provide different kinds of protection. Adopting the definitions by Doolan et al. (2009), we define protection to be objective evidence of a lower risk of clinical disease, indicated by the absence of fever, that is, the oral temperature does not exceed the threshold $37\,^{\circ }$C (Clark and Kruse 1990). *Anti-disease immunity* is conferred protection against clinical disease, which affects the overall risk and extent of morbidity associated with a given parasite density. *Anti-parasite immunity* is conferred protection against parasitemia, which affects the parasite density. *Premunition* provides protection against new infections by maintaining a generally asymptomatic parasitemia (Koch 1900; Sergent and Parrot 1935). In this article, we make use of a kind of *premunition* called naturally acquired immunity (NAI). As reported by Doolan et al. (2009), in holoendemic regions across sub-Saharan Africa, most people are continuously infected by *P. falciparum*, while the majority of infected adults rarely experience observable disease. This valid protection against infection is NAI corresponding to *P. falciparum*.

Mathematical models of malaria transmission have been studied by various authors; for a survey, we refer the reader to Mandal et al. (2011). Model formulations where partially immune humans are allowed to be infective have been studied by Ngwa and Shu (2000) and Roop-O et al. (2015). Both of these models accounted for partially immune humans in the recovered compartment. Asymptomatic malaria has also been previously modeled and studied. Of recent, an asymptomatic malaria model was introduced by Filipe et al. (2007). This model depends on a state-invariant control parameter $\phi$, which stands for the proportion of human infections that develop disease. After letting 1/*h* denote the mean latent period in humans, the progression rates from the exposed to the symptomatic and asymptomatic classes were defined to be the products $h\phi$ and $h(1-\phi )$, respectively. Additionally, asymptomatic humans were included in the recovered compartment.

In this article, we depart from the previous models of asymptomatic malaria by creating explicitly different compartments for symptomatic (*Y*) and asymptomatic (*A*) subjects, which in addition to susceptibles (*S*), exposed (*E*), and recovered (*R*) yields the acronym $SEY AR$. Another important point of departure with respect to previous models of asymptomatic malaria is that in the $SEY AR$ model (4), the progression rates corresponding to the symptomatic and asymptomatic human classes are nonlinear functions of the time-dependent exposed proportion. Therefore, the $SEY AR$ model does not fall into a sub-class of such models currently appearing in the literature. Moreover, we do not include the asymptomatic humans in the recovered compartment. This allows an effective isolation of the effect that asymptomatic carriers have on the disease transmission dynamics. Unlike other models appearing in the literature, the recovered human compartment studied in this article does not have an associated transmission probability, since it does not contribute to mosquito infection.

The derivation of the $SEY AR$ model (4) hinges on a specific decomposition of the infected human compartment into two mutually disjoint sub-compartments accounting for asymptomatic and symptomatic carriers. This decomposition is accomplished by making use of a nonlinear exposure-dependent NAI function, which is the solution to the initial-value problem (1) derived in Sect. 2. Although there is much in the literature, the proper inclusion of asymptomatic carriers into the epidemiological modeling of malaria warrants a formal mathematical understanding.

This manuscript provides a new malarial model accounting for asymptomatic human hosts in terms of the NAI of the population under consideration and is organized as follows: Sect. 2 presents the model formulation, Sect. 3.1 covers the issue of well-posedness of the initial-value problem and provides an analysis of the total population dynamics, Sect. 3.2 contains a rigorous study of the local asymptotic stability of disease-free equilibrium (DFE) for the model with a mathematical and epidemiological interpretation of the reproductive threshold, Sect. 4 introduces some modifications of the $SEY AR$ model along with their corresponding reproductive thresholds and addresses the impact of the asymptomatic class on the reproductive threshold of the original model, Sect. 5 is focused on nonlinear stability analysis and provides a classification parameter in which its size determines the type of bifurcation undergone by the dynamical system, Sect. 6 incorporates control measures into the dynamical system, Sect. 7.1 is focused on a sensitivity analysis of the reproductive threshold arising from the model, Sect. 7.2 consists of numerical results corresponding to the following three high transmission sites: Kaduna in Nigeria, Namawala in Tanzania, and Butelgut in Papua New Guinea, Sect. 8 consists of a summary of the results contained in Sects. 2–7.2 and a discussion regarding future direction and extensions, “Appendix A” contains formal proofs of the lemmas and theorems contained in Sects. 2–6, “Summary of Stability Theorems” of appendix is a summary of the main stability theorems used in the investigation of the local asymptotic stability of the equilibrium solutions studied in Sects. 3.2 and 5, and “Parameter Values” of appendix contains tables of numerical rates corresponding to the high transmission sites studied in Sect. 7.2.

## Methods: Model Formulation

The formulation of the $SEY AR$ model for the spread of malaria in the human and mosquito populations begins with dividing the total host–vector population into two compartments, denoted by $N_{{H}}(t)$ and $N_{{M}}(t)$, which stand for the total population sizes of the humans and mosquitoes, respectively, at a given time *t*. From this point on, whenever implied by the context of the discussion, the time *t* dependency is suppressed. Assuming a homogeneously mixed host population, we further decompose the compartments into the following five epidemiological classes: susceptible human *S*, exposed human *E*, symptomatic human *Y*, asymptomatic human *A*, and recovered human *R*, so that $N_{{H}}=S+E+Y+A+R$. For simplicity of exposition, the state variable is identified with its corresponding class. For example, when we are considering a human from class *A*, it is understood that *A* is a function and not a class, in general.

A point of departure from the usual $SEIR$ models, as studied by d’Onofrio (2002), Li and Muldowney (1995), Li et al. (1999), Roop-O et al. (2015) and Smith et al. (2001), resides in the mutually disjoint partitioning of the infected compartment into two sub-compartments, labeled asymptomatic *A* and symptomatic *Y*, i.e., $I=Y \cup A$ where $Y \cap A= \varnothing$. For the mosquito population, we have the following three classes: susceptible mosquito $M_{{S}}$, exposed mosquito $M_{{E}}$, and infected mosquito $M_{{I}}$. Accordingly, the total mosquito population is given by $N_{{M}}=M_{{S}}+M_{{E}}+M_{{I}}$.

As mentioned by Filipe et al. (2007), it is known that the infection rates between human and mosquito populations depend on numerous factors including the human biting rate of the mosquito $\sigma$ (which is the number of bites per mosquito), transmission probabilities (to be later defined), and the number of infectious and susceptible of each species involved. Furthermore, we assume that the average number of mosquito bites suffered by humans depends on the total sizes of their respective populations in the community. As a result, the number of bites per human is $\sigma \frac{N_{{M}}}{N_{{H}}}$. Therefore, the force of infection from mosquitoes to humans $\lambda _{ SE }$ is defined to be the product of the number of bites per human, the transmission probability $\beta _{{M}}$ from a mosquito in the class $M_{{I}}$ to a human in class *S*, and the probability that a mosquito is infectious $\frac{M_{{I}}}{N_{{M}}}$, i.e.,

$$\begin{aligned} \lambda _{ SE } =\omega _{M} = \sigma \frac{N_{{M}}}{N_{{H}}} \ \beta _{{M}} \ \frac{M_{{I}}}{N_{{M}}}=\sigma \beta _{{M}} \frac{M_{{I}}}{N_{{H}}}. \end{aligned}$$

Even though asymptomatic carriers might not get clinically ill, they still could harbor low levels of *gametocytes* in their bloodstreams and could to pass the infection onto mosquitoes (Vinetz and Gilman 2002). When a mosquito from class $M_{{S}}$ bites a human from class *Y*, the force of infection $\omega _{Y}$ is defined as the product of the number of bites per mosquito $\sigma$, the transmission probability $\beta _Y$ from a human in *Y* to a mosquito in $M_{{S}}$, and the probability that a human is in the symptomatic class $\frac{Y}{N_{{H}}}$. When a mosquito from the class $M_{{S}}$ bites a human from class *A*, the corresponding force of infection $\omega _{A}$ is the product of the number of bites per mosquito $\sigma$, the transmission probability $\beta _A$ from a human in *A* to a mosquito in $M_{{S}}$, and the probability that a human is in the asymptomatic class $\frac{A}{N_{{H}}}$. As pointed out by Laishram et al. (2012a), the parasites carried by asymptomatic hosts can be more infectious than those of symptomatic hosts. One could assume that a typical asymptomatic carrier has a higher NAI level than a symptomatic, so that $\beta _A \le \beta _Y$. Accordingly, the force of infection from humans to mosquitoes $V_{ SE }$ is defined to be the sum of the forces of infection corresponding to the humans in classes *Y* and *A*, i.e.,

$$\begin{aligned} \nu _{ SE }=\omega _{Y} + \omega _{A} =\sigma \Big ( \beta _Y \frac{Y}{N_{{H}}} +\beta _A \frac{A}{N_{{H}}}\Big ). \end{aligned}$$

Let $\nu _{{EI}}=\tau$, where $\tau$ is the reciprocal of the mean duration of the definitive host latent period.

At a given time $t \in {\mathbb {R}}_+$, an individual’s experience of malaria is dependent upon the degree of naturally acquired immunity that he or she has gained. Effective anti-parasitic immunity is achieved only after many frequent infections (Carter and Mendis 2002; James et al. 1920; Macdonald et al. 1957). This important epidemiological observation, in combination with the discussion regarding naturally acquired immunity (NAI) in Sect. 1, implies that the rate of progression $\lambda _{EA}$ from the exposed class *E* to asymptomatic class *A* depends on the proportion of human individuals receiving sufficient protection from the average NAI accumulated in the population with respect to natural exposure.

Let *u*(*t*) denote the proportion of the human population fully protected by NAI. Since naturally acquired immunity to the *Plasmodium* parasite is acquired and accumulates over time in response to frequent exposure, the rate that this proportion of protected individuals changes depends on the rate that the human population is being exposed, up to a threshold value. To uncover this exposure dependency, firstly let the lower and upper protected proportion thresholds be given by $u_{_{{\textit{low}}}}$  and $u_{_{{\textit{high}}}}$, respectively. It should be noted that $0\le u_{_{{\textit{low}}}}<u_{_{{\textit{high}}}}<1$.

Let $\varepsilon :=\frac{E}{N_{{H}}}$, upon assuming that the initial NAI protected proportion is given by the lower threshold $u(0):=u_{_{{\textit{low}}}}$, these epidemiological principles lead to the following initial-value problem (IVP) being posed

1 $$\begin{aligned} {\left\{ \begin{array}{ll} {\dot{u}}= (u_{_{{\textit{high}}}}- u) {\dot{\varepsilon }},\\ u(0)=u_{_{{\textit{low}}}}. \end{array}\right. } \end{aligned}$$

Notice that the time derivative on both sides of the differential equation (1) is an unusual formulation. It is explained by the fact that the rate of the human population fully protected by NAI ${\dot{u}}$ is dependent upon the rate that the population is exposed to the pathogen ${\dot{\varepsilon }}$. For example, if ${\dot{\varepsilon }}$ increases or decreases, then it stands to epidemiological reason that ${\dot{u}}$ should increase or decrease.

By making use of the integrating factor $L=e^{\int _{0}^t {\dot{\varepsilon }}(s)\mathrm{d}s}:=e^{\varepsilon -\varepsilon _0}$, it follows that

$$\begin{aligned} \dot{(Lu)}&= L{\dot{\varepsilon }} u_{_{{\textit{high}}}},\\ Lu&=u_{_{{\textit{low}}}}\,+\,(L-1)u_{_{{\textit{high}}}},\\ u&=L^{-1}u_{_{{\textit{low}}}} + (1-L^{-1})u_{_{{\textit{high}}}},\\ u&= e^{\varepsilon _0-\varepsilon } u_{_{{\textit{low}}}} + (1-e^{\varepsilon _0-\varepsilon })u_{_{{\textit{high}}}}.\\ \end{aligned}$$

Upon rearranging terms and invoking a slight abuse of notation, to emphasize the exposure $\varepsilon$ dependency of *u*, the solution is represented by the following equation:

2 $$\begin{aligned} u(\varepsilon )=e^{\varepsilon _0-\varepsilon }(u_{_{{\textit{low}}}}\,-u_{_{{\textit{high}}}}) + u_{_{{\textit{high}}}}. \end{aligned}$$

In the above, the symbol $\varepsilon _0:=\varepsilon (0)$ stands for the initial exposed proportion of the human host population. The function $N_{{H}}$ is positive for all $t \in {\mathbb {R}}_+$; thus, it directly follows that $\varepsilon \in C^1_b({\mathbb {R}}_+).$ In general, care should be taken to ensure that the progression rate is mathematically well defined and epidemiologically sensible, i.e., a singularity should not arise and it should be non-negative. Provided that ${\mathbf {x}}$ is such that $\varepsilon \in C^1({\mathbb {R}}_+)$, then clearly the progression rate will not experience a singularity.

Besides the trivial singularity issue covered above, care must be taken to ensure the non-negativity of the nonlinear progression rate *u*. As a result, the solution to the IVP (1) implicitly imposes an additional constraint upon the initial data of the $SEY AR$ model (4). The constraint is listed below in Lemma 1, which is proven in “Appendix.”

### Theorem 1

(Initial data constraint for the SEYAR model) Let $\vartheta$ be defined as follows:

3 $$\begin{aligned} \vartheta :=\ln \left( \frac{u_{_{{\textit{high}}}}}{u_{_{{\textit{high}}}}-u_{_{{\textit{low}}}}}\right) . \end{aligned}$$

To ensure the non-negativity of *u*, the initial data are required to satisfy the inequality $E_0 \le \vartheta N_0$.

Let the eight-dimensional vector of functions ${\mathbf {x}}=\left( S,E,Y,A,R,M_{{S}},M_{{E}},M_{{I}}\right) ^T$ be such that $\varepsilon \in C^1({\mathbb {R}}_+)$ and $\vartheta$ be defined as in Theorem 1. The nonlinear progression rate *u* is well defined in a mathematical and epidemiological sense.

Define $\lambda _{EA}=\gamma u(\varepsilon )$ where $\gamma$ is the reciprocal of the mean duration of the human latent period. It is a direct consequence that $\lambda _{EY}=\gamma (1-u(\varepsilon ))$, so that $\lambda _{EA}+\lambda _{EY}=\gamma$. Since the naturally acquired immune proportion will grow in response to exposure, the rate of progression from the exposed class *E* to asymptomatic class *A* should increase, warranting the choice of $\lambda _{EA}$. Furthermore, as the exposure rate increases, $\lambda _{EA}$ will eventually be maximized. This is consistent with the observation that the average amount of asymptomatic human hosts in a population should increase after frequent exposure over a sufficient time period. When the exposed proportion is equal to zero over a prescribed time interval, it follows that $u=e^{\varepsilon _0}(u_{_{{\textit{low}}}}\,-u_{_{{\textit{high}}}}) + u_{_{{\textit{high}}}}$ over the interval. This quantity is a sum consisting of the upper threshold and a negative scaled difference of the lower and upper thresholds. If this infimum is achieved, then the progression rate $\lambda _{EA}$ will be minimal. This is due to the fact that if there is little exposure, then there is little NAI developed in the population, so that the rate of progression from *E* to *Y* will be maximal. Moreover, the progression rate from *E* to *Y* should decrease as the exposure rate increases.

If the population under consideration is free of malaria, then no immunity should be present and it is possible that $u_{_{{\textit{high}}}}=0$. In this scenario, we set $u_{_{{\textit{low}}}} =0$. As a result, $u=0$ for all time and it follows that $\lambda _{EA}=0$. Thus, the progression rate from the exposed human class to the asymptomatic class is nullified. However, as the purpose of this article is to model asymptomatic malaria, it is assumed that malaria is present in the population.

**Fig. 1:**
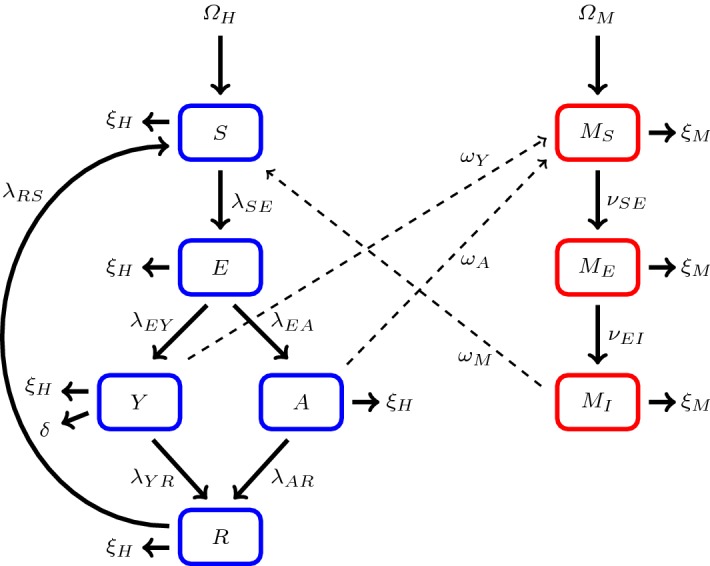
This figure is a schematic diagram of a malaria model including an asymptomatic compartment. The solid lines represent progression from one compartment to the next, while the dotted stand for the human–mosquito interaction. Humans enter the susceptible compartment either through birth or migration and then progress through each additional compartment subject to the rates described above (Color figure online)

These assumptions give rise to the following $SEY AR$ model IVP (4), depicted in Fig. 1, describing the dynamics of malaria disease transmission in the human and mosquito populations:

4 $$\begin{aligned} {\left\{ \begin{array}{ll} {\dot{S}} =\varOmega _{{H}} + \lambda _{RS} R - \left( \sigma \beta _{{M}} \frac{M_{{I}}}{N_{{H}}} + \xi _{{H}} \right) S,\\ {\dot{E}} =\sigma \beta _{{M}} \frac{M_{{I}}}{N_{{H}}}S - (\gamma +\xi _{{H}})E,\\ {\dot{Y}} =\gamma (1-u(\varepsilon )) E - \left( \xi _{{H}} +\delta + \lambda _{YR}\right) Y,\\ {\dot{A}} =\gamma u(\varepsilon ) E - \left( \lambda _{AR}+\xi _{{H}}\right) A,\\ {\dot{R}} =\lambda _{AR}A + \lambda _{YR}Y - \left( \lambda _{RS} +\xi _{{H}}\right) R, \\ \dot{M_{{S}}} =\varOmega _{{M}} - \left( \xi _{{M}} + \sigma \beta _Y \frac{Y}{N_{{H}}} +\sigma \beta _A \frac{A}{N_{{H}}} \right) M_{{S}}, \\ \dot{M_{{E}}} =\sigma \left( \beta _Y \frac{Y}{N_{{H}}} +\beta _A \frac{A}{N_{{H}}}\right) M_{{S}} - \left( \xi _{{M}} + \tau \right) M_{{E}},\\ \dot{M_{{I}}} =\tau M_{{E}}- \xi _{{M}} M_{{I}}, \\ \left( S_0,E_0,Y_0,A_0,R_0,M_{S_0},M_{E_0},M_{I_0}\right) ^T\in {\mathbb {R}}^8_+ \text { such that } \varepsilon _0 \le \vartheta , \end{array}\right. } \end{aligned}$$

where *u* and $\vartheta$ are defined by Eqs. (2) and (3), respectively. For convenience, the model parameters are summarized in Table 1. All of the parameters are strictly positive, with the exception for the disease-induced death rate $\delta$, which is allowed to be non-negative. The naturally acquired immune proportion will increase or decrease, depending on the rate that the human population is being exposed. Hypothetically, as the rate of exposure increases or decreases, this proportion should grow or shrink up to a threshold value. This epidemiological behavior is quantified by the solution to the IVP (1). Assume $u_{_{{\textit{low}}}}=0.5$, as above, and that it is possible for at most ninety percent of the population to acquire sufficient protection through means of natural exposure, i.e., $u_{_{{\textit{high}}}}=0.9$. Then, the initial exposed proportion $\varepsilon _0$ can be assumed to take any value in the interval $\left[ 0,\ln \left( \frac{u_{_{{\textit{high}}}}}{u_{_{{\textit{high}}}}-u_{_{{\textit{low}}}}}\right) \right] =[0,\ln \left( \frac{9}{4}\right) ] \approx [0,0.81]$. In other words, one can assume at most $81\%$ of the human population to be initially exposed. On the other hand, if one modifies the above assumptions so that the initial naturally acquired immune proportion is $u_{_{{\textit{low}}}}=0.1$, then $\varepsilon _0 \in [0,\ln \left( \frac{9}{8}\right) ] \approx [0,0.12]$, so that at most $12\%$ of the human population can be assumed to be initially exposed.

**Table 1:** Model parameters

Parameter	Description	Dimension
$$\varOmega _{{H}}$$	Recruitment rate of humans	humans $$\times $$$$\text {time}^{-1}$$
$$\varOmega _{{M}}$$	Recruitment rate of mosquitoes	mosquitoes $$\times $$$$\text {time}^{-1}$$
$\xi _{{H}}$	Natural mortality rate of human	$\text {time}^{-1}$
$\xi _{{M}}$	Natural mortality rate of mosquito	$\text {time}^{-1}$
$\beta _{A}$	Probability of disease transmission from asymptomatic human to a susceptible mosquito	n/a
$\beta _Y$	Probability of disease transmission from symptomatic human to a susceptible mosquito	n/a
$\beta _{{M}}$	Probability of disease transmission from infected mosquito to susceptible human	n/a
$\gamma$	The intermediate host mean latent period	$\text {time}^{-1}$
$\tau$	The definitive host mean latent period	$\text {time}^{-1}$
$\delta$	Disease-induced death rate for humans	$\text {time}^{-1}$
$\sigma$	Human biting rate of mosquito	$\text {time}^{-1}$
$\lambda _{AR}$	Asymptomatic human recovery rate	$\text {time}^{-1}$
$\lambda _{YR}$	Symptomatic human recovery rate	$\text {time}^{-1}$
$\lambda _{RS}$	Temporary immunity loss rate in humans	$\text {time}^{-1}$
$u(\varepsilon )$	Exposure-dependent NAI protected proportion	n/a
$u_{_{{\textit{low}}}}$,$u_{_{{\textit{high}}}}$	Lower and upper NAI protected thresholds	n/a

## Model Analysis

### Well-Posedness and Feasible Region

Although assuming that $\varPhi \in C^1$ provides sufficient regularity to ensure that system (5) is well posed, this work is primarily concerned with the stability of the system near equilibrium points. This requires additional regularity assumptions on the vector field $\varPhi$ in order to invoke a variation of the center manifold theorem, proven by Castillo-Chavez and Song (2004). Consequently, from now on, it is necessary to assume that $\varPhi \in C^2\subset C^1$, that is, it is at least twice continuously differentiable. Moreover, to be reasonable in an epidemiological sense, the functions under consideration should posses a bounded first derivative, that is, they should be members of the class $C^1_b({\mathbb {R}}_+)$. From this point on, the model is studied in the more regular (smaller) function space $C^2({\mathbb {R}}^8_+) \cap C^1_b({\mathbb {R}}^8_+)$. In light of the mathematical and epidemiological well-posedness of the IVP (1) and structure of the underlying vector field, the additional $C^2$ regularity will be inherited by the solution ${\mathbf {x}}$.

To this end, let ${\mathbf {x}}=\left( S,E,Y,A,R,M_{{S}},M_{{E}},M_{{I}}\right) ^T$, so that $x_i$ is the $i\text {th}$ component of the eight-dimensional vector ${\mathbf {x}} \in C^2({\mathbb {R}}^8_+) \cap C^1_b({\mathbb {R}}^8_+)$ and we rewrite (4) in the following compact form:

5 $$\begin{aligned} {\left\{ \begin{array}{ll} \dot{{\mathbf {x}}}(t)=\varPhi \left( {\mathbf {x}}(t)\right) , \\ {\mathbf {x}}(0)={\mathbf {x}}_0. \end{array}\right. } \end{aligned}$$

#### Theorem 2

(Existence theory of the $SEY AR$ Model) There exists a sufficiently regular unique solution ${\mathbf {x}}$ to the SEYAR model IVP (5) that can be continued to a maximal time interval. Additionally, ${\mathbf {x}}$ depends continuously on the initial data ${\mathbf {x}}_0$ and model parameters involved.

For the proof of the above theorem, the reader is referred to “Proof of Theorem 2 (See Page 11)” of appendix. The dynamics of the total population are given by the following decoupled system:

6 $$\begin{aligned} {\left\{ \begin{array}{ll} {\dot{N}}_{{H}} = \varOmega _{{H}}-\xi _{{H}} N_{{H}}-\delta Y,\\ {\dot{N}}_{{M}} =\varOmega _{{M}}-\xi _{{M}} N_{{M}}.\\ \end{array}\right. } \end{aligned}$$

Due to the non-homogeneous term $\delta Y$, the asymptotic behavior of the human population is more delicate. For the human population, we have the following theorem which provides a tighter lower bound on the attracting region for the model. In the absence of infection, the long-time behavior of $N_{{H}}$ is trivial, that is, the total human population converges to the equilibrium population density, as in the mosquito population. In the case of an infectious disease with disease-induced death rate as in Eq. (6), one would expect the population to converge to a smaller quantity, as there is a disease-induced death rate $\delta$ adding to the natural death rate $\xi _{{H}}$. The long-time population size will be smaller, as it has to account for the additional disease-induced deaths suffered by symptomatic individuals. Listed below is the theorem concerning the feasible region of model (6).

#### Theorem 3

(Feasible region of the $SEY AR$ model) Let $\left( N_{{H}}, N_{{M}}\right)$ be the solution of system (6) emanating from Theorem 2, with corresponding initial data $\left( N_{{H}}(0), N_{{M}}(0)\right) \in {\mathbb {R}}^2_+$. Define the following compact sub-space

$$\begin{aligned} \varGamma :=\left\{ {\mathbf {x}} \in C^2({\mathbb {R}}^8_+) \cap C^1_b({\mathbb {R}}^8_+){:}\,N_{{H}} \in \left[ \alpha ,\frac{\varOmega _{{H}}}{\xi _{{H}}} \right] , N_{{M}} = \frac{\varOmega _{{M}}}{\xi _{{M}}}\right\} , \end{aligned}$$

where $\alpha := \max \left( 0, \frac{\varOmega _{{H}} - \delta \Vert Y \Vert _\infty }{\xi _{{H}}}\right)$. Then, $\varGamma$ is a forward invariant attractor for system (6).

For the proof of the above theorem, the reader is referred to “Proof of Theorem 3 (See Page 12)” of appendix. Mathematically speaking, Theorem 3 reduces the complexity of the analysis involved regarding the long-term dynamics of the system by allowing the replacement of a potentially unbounded (epidemiologically unreasonable) space with the smaller (epidemiologically reasonable) compact sub-space $\varGamma$. If we let $L_t=e^{\small {\xi _{{H}} t}}$ denote an exponential multiplier, then the solution for the total dynamics of the human population is given by

$$\begin{aligned} N_{{H}}(t)=L_{-t}N_{{H}}(0)+ \frac{\varOmega _{{H}} }{\xi _{{H}}}\left( 1-L_{-t}\right) -\delta L_{-t} *Y(t). \end{aligned}$$

The instantaneous rate of occurrence of death, i.e., the force of mortality $\xi _{{H}}$, is assumed to be constant. As a result, the probabilities of living and dying up to *t* days are given by $e^{-\xi _{{H}}t}$ and $\left( 1-e^{-\xi _{{H}}t}\right)$, respectively. Assuming that $N_{{H}}(0)=0$, the above solution says that the total population $N_{{H}}(t)$ at time *t* is given by the weighted product of the carrying capacity $\frac{\varOmega _{{H}} }{\xi _{{H}}}$ and the distribution of humans that are left after those that have died due to natural causes $\left( 1-e^{\small {-\xi _{{H}} t}}\right)$, minus a weighted average of humans that have died due to symptomatic infections. The later quantity is captured by the non-homogeneous forcing term $-\,\delta L_{-t} *Y(t)$, given by a convolution with the inverse multiplier. Since convolution is a smoothing operation, this emphasizes the fact that we are subtracting a “smoothing average” over past time of the humans which have died from symptomatic infections. A straightforward calculation yields the following differential inequality:

$$\begin{aligned} {\dot{N}}_{{H}} \le 0,\quad \text {if} \quad N_{{H}} \ge \frac{\varOmega _{{H}}}{\xi _{{H}}}. \end{aligned}$$

From an epidemiological view point, the above inequalities imply that if the total population $N_{{H}}$ breaches its carrying capacity, then the weighted average of fatal symptomatic infections must increase to stabilize the population back to a healthy level.

In an epidemiological setting, one can define the term $\delta \Vert Y \Vert _\infty$ to be the potential maximum disease-related death of the human population. If $\varOmega _{{H}} > \delta \Vert Y \Vert _\infty$, then it directly follows that $\alpha =\frac{\varOmega _{{H}} - \delta \Vert Y \Vert _\infty }{\xi _{{H}}}$. In this case, the above theorem provides a tighter lower bound for the feasible region corresponding to the dynamical system (6). In practice, the disease-induced death rate for humans $\delta$ is sufficiently small (and in some cases negligible), so that this inequality is satisfied.

In the previous variants of malaria models appearing in the literature, e.g., SIR, SEIR, SEIRS, etc., the quantity 0 is listed as the lower bound; however, this is unreasonable since the populations under consideration usually do not go extinct, unless $\varOmega _{{H}}=\delta \Vert Y \Vert _\infty$. If the maximum impact the disease is capable of having on the population is less than the recruitment rate, then their will always be accumulation over long time. For other members of the SIR model class, the lower bound would be the same except with *Y* replaced by *I*. In the case of an infectious disease, there will be additional disease-induced deaths, so that the total human population will not converge to the equilibrium population density.

### Reproductive Threshold and Disease-Free Equilibrium

This section is focused on deriving a threshold value that characterizes the local asymptotic stability of the underlying dynamical system (4). This value, called the reproductive threshold, provides a way to estimate the reduction in transmission intensity required to eliminate malaria through vector-based control (Smith et al. 2007). The basic reproductive number ${\mathcal {R}}_0$ corresponding to a given model is a threshold value which represents the average amount of new infections produced by a typical infectious individual in a completely susceptible population, at a disease-free equilibrium. This quantity is equal to the reproductive threshold for a class of simplified population models.

Disease-free equilibrium (DFE) points are solutions of a dynamical system corresponding to the case where no disease is present in the population. Define the diseased classes to be $E,Y,A,M_{{E}}$, and $M_{{I}}$. Notice that *R* is not considered to be a diseased class, as the asymptomatic class *A* has been effectively removed, cf. Roop-O et al. (2015). As a result, individuals in the *R* compartment are considered to be temporarily immune, but not infectious. After determining the DFE of system (4), this threshold value is used to address its local asymptotic stability. Upon equating the right-hand side of (4) to zero and solving, we arrive at the following unique DFE:

$$\begin{aligned} {\mathbf {x}}_{\small {{dfe}}}=\left( \frac{\varOmega _{{H}}}{\xi _{{H}}},0,0,0,0,\frac{\varOmega _{{M}}}{\xi _{{M}}},0,0\right) ^T. \end{aligned}$$

#### Lemma 1

(Local asymptotic stability of the DFE for the $SEY AR$ model) Define the following quantity:

7 $$\begin{aligned} {\mathcal {R}}_0 :=\sqrt{\frac{\sigma ^2 \tau \gamma \varOmega _{{M}} \xi _{{H}} \beta _{{M}}}{\xi _{{M}}^2(\gamma + \xi _{{H}})(\tau + \xi _{{M}})\varOmega _{{H}}} \left( \frac{\beta _AU_{_{{\textit{low}}}} }{\lambda _{AR} + \xi _{{H}}} - \frac{\beta _Y(U_{_{{\textit{low}}}}\,-1)}{\lambda _{YR} + \xi _{{H}} + \delta } \right) }, \end{aligned}$$

where $U_{_{{\textit{low}}}}:=e^{\varepsilon _0}(u_{_{{\textit{low}}}}\,-u_{_{{\textit{high}}}}) + u_{_{{\textit{high}}}}$. Then, the DFE ${\mathbf {x}}_{\small {{dfe}}}$ for the SEYAR model (4) is locally asymptotically stable provided that ${\mathcal {R}}_0<1$ and unstable if ${\mathcal {R}}_0>1$ in $\varGamma$.

For the proof of Lemma 1, the reader is referred to “Proof of Corollary 1 (See Page 18)” of appendix. A verification of the reproductive threshold ${\mathcal {R}}_0$ (7) is provided in the electronic supplementary material. Lemma 1 is proven by utilizing the next-generation method developed by Van den Driessche and Watmough (2002). The threshold value (7) has major epidemiological implications on the underlying dynamical system (4). To gain a deeper insight into the qualitative information encoded in this important quantity, we decompose it in the form of an epidemiological meaningful product in order to analyze each factors contribution:

$$\begin{aligned} {\mathcal {R}}_0&=\sigma \sqrt{\frac{\varOmega _{{M}}}{\varOmega _{{H}}}}\sqrt{\frac{\tau }{\tau + \xi _{{M}}}}\sqrt{\frac{\gamma }{\gamma + \xi _{{H}}}}\sqrt{\frac{\xi _{{H}}}{\xi _{{M}}}}\sqrt{\frac{\beta _{{M}}}{\xi _{{M}}}}\sqrt{\frac{\beta _AU_{_{{\textit{low}}}} }{\lambda _{AR} + \xi _{{H}}} - \frac{\beta _Y(U_{_{{\textit{low}}}}\,-1)}{\lambda _{YR} + \xi _{{H}} + \delta }}, \\&:=\sigma \prod _{i=1}^6 \sqrt{r_i}. \end{aligned}$$

Due to the above lemma and in an epidemiological setting, it is desirable to have the reproductive threshold below unity. Listed below is the size contribution and biological description for each of the factors. The first factor is $\sigma$, which stands for the human biting rate. This factor is usually much less than unity due to the fact that female anopheline mosquitoes generally transmit fewer than 100 *sporozoites* per bite (Ponnudurai et al. 1991). As malaria is a mosquito borne disease, the agent *Plasmodium* will spread at a much slower rate, provided less vectors are introducing it into human hosts. Owning to the monotonicity of the square root function, it is sufficient focus on the size of each $r_i$.

i The term $r_1=\frac{\varOmega _{{M}}}{\varOmega _{{H}}}$ is the ratio of the mosquito and human recruitment rates. The population density of anopheles mosquitoes is a function of proximity to breeding sites. A study in the urban settings of Dakhar in 1998 (Trape et al. 1992) reports an average mosquito population density of 10.4 mosquito/room in general, and specifically 1.6 *Anopheles*/room during the rainy season; these counts correspond to mosquitoes collected after indoor pyrethrum spray and do not account for outdoor mosquito activity. These numbers seem to indicate that the population of *Anopheles* mosquitoes is larger than the population of humans in that urban setting. Likewise, a study in a rural area of Sudan in a $4\,\mathrm{km}^2$ area inhabited by approximately 800 people reported a mosquito population in the range 135,000–330,000 mosquitoes (Costantini et al. 1996). Combined, these studies suggest that in an endemic site, the quotient $r_1$ is greater than unity. As the human and mosquito recruitment rates rank high on the sensitivity hierarchy of many epidemic models appearing in the literature, it is no surprise that this term is problematic with respect to the overall size of the threshold.
ii The term $r_2=\frac{\tau }{\tau + \xi _{{M}}}$ is the reciprocal of the mosquito latent period $\tau$ divided by itself plus the mosquito mortality rate $\xi _{{M}}$. This quantity is bounded above by one and is monotonically decreasing with respect to $\xi _{{M}}$. It follows that the larger the mosquito death rate is, the smaller $r_2$ will be.
iii The first fully human-dependent term $r_3=\frac{\gamma }{\gamma + \xi _{{H}}}$ is comprised of the reciprocal of the human latent $\gamma$ period divided by itself plus the human mortality rate $\xi _{{H}}$. As in the case of $r_2$, this quantity is always less than one and monotonically decreases with respect to the human mortality rate. This is consistent with the fact that the fewer hosts there are for the parasite to invade, the less infections will arise. However, since increasing $\xi _{{H}}$ is not practical, this terms offers no control over ${\mathcal {R}}_0$.
iv The term $r_4=\frac{\xi _{{H}}}{\xi _{{M}}}$ is the ratio of the human and mosquito death rates. This particular quantity is always less than one as the mosquito death rate is much higher than the human death rate.
v The term $r_5=\frac{\beta _{{M}}}{\xi _{{M}}}$ is the ratio of the mosquito-to-human transmission probability $\beta _{{M}}$ and the mortality rate of the mosquito population $\xi _{{M}}$. This quantity will be less than one provided $\beta _{{M}}<\xi _{{M}}$. In the case of a population with a relatively high vector transmission probability, the vector death rate must be large enough to make $r_5$ less than one. This implies a restriction on the size of $\beta _{{M}}$. It will be shown in Sect. 5 that if $\beta _{{M}}$ breaches a certain threshold, then sub-threshold endemic equilibria can emerge.
vi The second fully human-dependent term is given by the following equation:
  $$\begin{aligned} r_6=\frac{\beta _AU_{_{{\textit{low}}}}}{\lambda _{AR} + \xi _{{H}}} - \frac{\beta _Y(U_{_{{\textit{low}}}}\,-1)}{\lambda _{YR} + \xi _{{H}} + \delta }. \end{aligned}$$
  The quantity $r_6$ is a difference of ratios consisting of asymptomatic and symptomatic vital dynamics, along with transmission, recovery, and disease-induced death rates, weighted with a distribution consisting of the lower and upper NAI-rate threshold of the human population scaled by an exponentiation of the initial exposure rate. For simplicity of exposition, we assume that the initial exposed proportion is zero, i.e., $\varepsilon _0=0$, so that $U_{_{{\textit{low}}}}=u_{_{{\textit{low}}}}$. If the initial exposed proportion is not equal to zero, then initially, $U_{_{{\textit{low}}}}<u_{_{{\textit{low}}}}$ and the reproductive threshold will be larger under parameter configurations to be specified shortly. Under such a configuration, upon initial exposure, there will be more symptomatic individuals, but as exposure increases, less humans will die as naturally acquired immunity will begin to develop in the overall population. However, the following discussion is unaffected by this minor detail. As a result, the human-dependent factor is taken to be:
  $$\begin{aligned} r_6=\frac{u_{_{{\textit{low}}}} \beta _A}{\lambda _{AR} + \xi _{{H}}} - \frac{(u_{_{{\textit{low}}}}\,-1) \beta _Y}{\lambda _{YR} + \xi _{{H}} + \delta }. \end{aligned}$$
  Let the low and high thresholds be such that $u_{_{{\textit{low}}}}>0$ and $u_{_{{\textit{high}}}}<1$, respectively, and define the following compact sub-interval $T_u := [u_{_{{\textit{low}}}},u_{_{{\textit{high}}}}] \subset (0,1)$ consisting of various NAI protected proportions corresponding to a given population. When subjected to a certain parameter restriction, the sizes of the quantities $u_{_{{\textit{low}}}}=u(0)$ and $r_6$ are inversely related, i.e., the larger $u_{_{{\textit{low}}}}$ is, the smaller $r_6$ will be, resulting in a relatively smaller ${\mathcal {R}}_0$. Hence, an additional way to control the size of ${\mathcal {R}}_0$ arises, provided the parameters are such that this inequality restriction, to be mentioned below, holds. However, as we will see, if the parameters are such that this inequality reversed, then they are directly related.
  Let $C_0:=\sigma \prod _{i=1}^5 \sqrt{r_i}$, $C_1:=\frac{\beta _A}{\lambda _{AR} + \xi _{{H}}}$, and $C_2:=\frac{\beta _Y}{\lambda _{YR} + \xi _{{H}} + \delta }$ and define $T \subset {\mathbb {R}}^+ \cup \{+\infty \}$ to be an ordered subset of the nonnegative extended real numbers. In addition, let *u* solve Eq. (2) and denote $\{u(t) \in T_u| t \in T \}$ to be the set of NAI protected proportions experienced by a population over a prescribed, possibly infinite, time interval indexed with *t* and consider the following function:
  $$\begin{aligned} {\mathcal {R}}_0(u(t)) :=C_0 \sqrt{(C_1-C_2) u(t)+ C_2}. \end{aligned}$$
  Subject to a given parameter configuration, the type of monotonicity obeyed by ${\mathcal {R}}_0(u(t))$ is dependent on the sign of the nonzero combination of parameters $C_1-C_2$. These observations motivate the following definition which classifies the configuration space of the model based on how the asymptotic dynamics of the corresponding population responds with respect to the NAI accumulated in response to the rate of exposure.

#### Definition 1

(*Configuration space*) The $SEY AR$ model (4) is said to possess a *Y-dominant configuration* if $C_1-C_2>0$ and an *A-dominant configuration*, provided that $C_1-C_2<0$. Upon the trivial case that $C_1-C_2=0$, the system is said to have a *null-configuration*. Additionally, we refer to a system possessing such a configuration as either *A*-, *Y*-, or *null-configured*. A given human population is called *A*-,*Y*-, or *null-dominant*, provided that its corresponding configuration is.

Although $r_6$ is always positive, the combination of parameters $C_1-C_2$ need not be. The positiveness of $r_6$ is a result of how the transmission probabilities are weighted by the NAI protected proportion. If the system is *null-configured*, then ${\mathcal {R}}_0(u(t)) :=C_0 \sqrt{C_2}$ and it is locally asymptotically stable, provided that $C_0<1$ and $\beta _Y<\lambda _{YR}+\xi _{{H}}+\delta$, i.e., a high vector death rate, and the symptomatics are recovering or dying out at a faster rate than they are transmitting. Consider the case of an *A-dominant configuration*, i.e.,

8 $$\begin{aligned} \frac{\beta _A}{\beta _Y} < \frac{\lambda _{AR} + \xi _{{H}}}{\lambda _{YR} + \xi _{{H}} + \delta }. \end{aligned}$$

As mentioned in Sect. 2, in the formulation of the $SEY AR$ model (4), we do not assume an ordering on the asymptomatic and symptomatic transmission probabilities $\beta _A$ and $\beta _Y$, respectively. However, provided that asymptomatic carriers transmit at a lower rate than that of symptomatic, the left-hand side of the above inequality is less than unity. Moreover, if asymptomatic individuals recover faster than symptomatic, provided the disease-induced death rate $\delta$ and recovery rates are such that $\lambda _{AR}>\lambda _{YR} + \delta$, then the right-hand side of the above inequality is greater than unity and inequality (8) is satisfied. In an epidemiological setting, such a configuration corresponds to holoendemic regions across sub-Saharan Africa. In such regions, the majority of people are continuously infected by *P. falciparum*, but only a small proportion display clinical symptoms (Águas et al. 2008). The high level of naturally acquired immunity present in the population allows them to live their daily lives feeling healthy despite a relatively high blood-parasite density (Doolan et al. 2009).

Analytically speaking, in the case of an *A-dominant configuration*, provided that the mosquito mortality rate $\xi _{{M}}$ can be made large enough so that the term $C_0$ compromised of fractional multipliers is sufficiently small, then ${\mathcal {R}}_0(u_t)$ achieves its maximum ${\mathcal {R}}_0(u_{_{{\textit{low}}}})$ at the low NAI threshold $u_{_{{\textit{low}}}}$ and decreases to its infimum as the high NAI threshold $u_{_{{\textit{high}}}}$ is approached. Moreover, since the ordered set *T* is a subset of a separable metric space, we can extract an ordered countable subset and form the partition $\{u_{_{{\textit{low}}}}=u(t_1)<\cdots<u(t_n)<u(t_{n+1})<\cdots \}$ of the compact sub-interval $T_u$. In this case, the corresponding values of ${\mathcal {R}}_0(u(t))$ obey the following descending order $\{{\mathcal {R}}_0(u_{_{{\textit{low}}}})>\cdots>{\mathcal {R}}_0(u(t_n))>{\mathcal {R}}_0(u(t_{n+1}))>\cdots \}$. This is consistent with the fact that as a given population acquires natural immunity through exposure, the disease will start to spread at a slower rate. Conversely, if the system has a *Y-dominant configuration*, i.e., $C_1-C_2>0$, then the monotonicity is reversed.

In mathematical terminology, provided $\sigma <1$, one can always find a large enough $\xi _{{M}}$ to make $C_0<1$. In this case, the factor that will cause ${\mathcal {R}}_0$ to breach unity is $r_6$. From an epidemiological standpoint, regardless of the size of vector transmission probability $\beta _{{M}}$ or human biting rate $\sigma$, if enough mosquitoes are dying to significantly slow the disease transmission dynamics, then $C_0<1$ and, as a result, the size of the reproductive threshold ${\mathcal {R}}_0$ will be determined by $r_6$ which depends on the human immune systems response to the *Plasmodium* parasite. This attests to the fact that in such vector transmitted diseases, the reproductive threshold will be lower in response to vector elimination up to a point and the factor allowing the disease to persist under such low vector activity lies in the intricate relationship between the parasite and host. This delicate relationship is captured by the term $r_6$.

## Variations of the $SEY AR$ Model and the Impact of the Asymptomatic Class

This section is primarily concerned with introducing some modifications of the $SEY AR$ model and their corresponding reproductive thresholds.

### Elimination of the *R* Compartment in the *SEYAR* Model

As mentioned earlier, premunition refers to instances when infected hosts carry low parasitemias that confer immunity to the symptoms of the disease. When these individuals are infectious, they are able to transmit the parasite to the mosquitoes; thus, they belong to the *A* compartment. In addition to showing no symptoms, some individuals exhibiting premunition are unable to transmit the parasite; these hosts are considered part of the *R* compartment. We consider next the scenario when sterilizing immunity via premunition is negligible, thus eliminating the need for an *R* compartment.

**Fig. 2:**
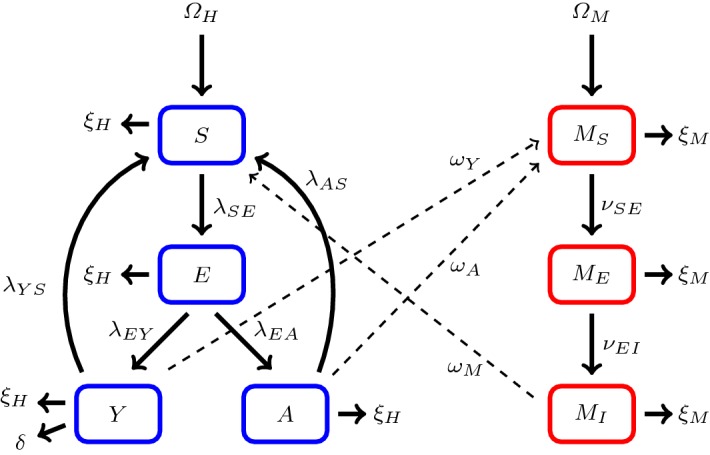
This figure is a schematic diagram of an asymptomatic malaria model without a recovered compartment. The solid lines represent progression from one compartment to the next, while the dotted stand for the human–mosquito interaction. Humans enter the susceptible compartment either through birth or migration and then progress through each additional compartment subject to the rates described above (Color figure online)

#### Corollary 1

(Local asymptotic stability of the DFE for the $SEY AR$ model without an *R* compartment) The reproductive threshold of the dynamical system corresponding to Fig. 2 is structurally the same as that of dynamical system corresponding to Fig. 1. The only difference resides in the relabeling of the terms $\lambda _{AR}$, $\lambda _{YR}$ to $\lambda _{AS}$, $\lambda _{YS}$, respectively.

A verification of Corollary 1 is provided in the electronic supplementary material. For the proof, the reader is referred to “Proof of Corollary 1 (See Page 18)” of appendix.

### Including Relapse Rates in the $SEYAR$ Model

During the course of *P. vivax* and *P. ovale* human infections, a number of *sporozoites* stay in the *hepatocytes* in a dormant stage for a variable amount of time ranging from weeks to years. This stage of infection is known as the *hypnozoite* stage and is not observed in *P. falciparum*, *P. malariae*, or *P. knowlesi*. It is unclear what causes a hypnozoite to become active, but when it does, it causes a relapse of the disease.

**Fig. 3:**
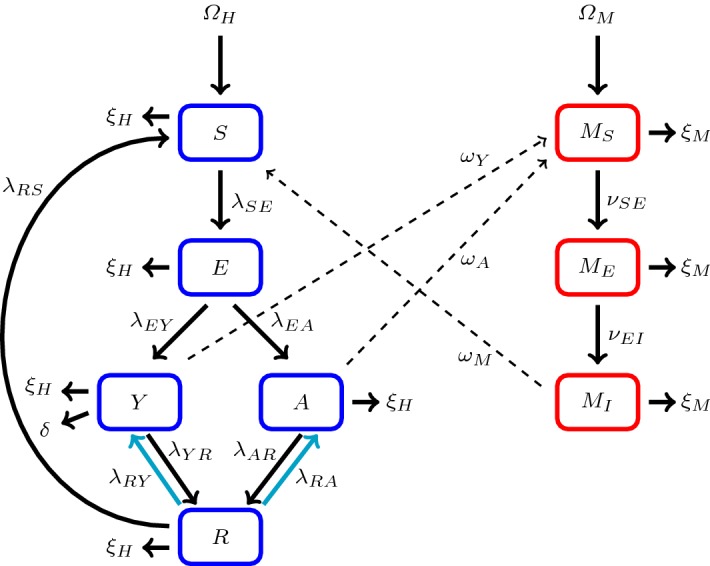
This figure is a schematic diagram of the $SEY AR$ malaria model including relapse rates. The solid lines represent progression from one compartment to the next, while the dotted stand for the human–mosquito interaction. Humans enter the susceptible compartment through recovery, birth, or migration and then progress through each additional compartment subject to the rates described above. The new solid lines have been highlighted to emphasize the model modification (Color figure online)

In order to properly account for such biological behavior, relapse rates are included into the $SEYAR$ model. The asymptomatic and symptomatic human relapse rates are denoted by $\lambda _{RA}$ and $\lambda _{RY}$, respectively. This slight adjustment yields the *P. vivax* and *P. ovale* version of the $SEY AR$ model shown in Fig. 3.

The model parameters and initial data restrictions are the same as in the original model. Moreover, the results of the mathematical analysis concerning the issues of well-posedness and nonlinear stability are similar to those of the original model. For example, the results regarding the LAS of the DFE for the above model variation are exactly the same as in the original model.

#### Corollary 2

(Local asymptotic stability of the DFE for the $SEY AR$ model including relapse rates) The dynamical system corresponding to Fig. 3 has the same reproductive threshold as the original dynamical system corresponding to Fig. 1.

A verification of Corollary 2 is provided in the electronic supplementary material. For the proof, the reader is referred to “Proof of Corollary 2 (See Page 20)” of appendix.

Therefore, the modified system has the same reproductive threshold as the original. This is due to the fact that the sub-matrices of the Jacobian *F* and *V*, resulting from the block matrix partitioning technique utilized in the next-generation method, are unaffected by this minor modification. The results regarding the LAS of the DFE corresponding to the new system are inherited from the original.

The above modification attests to the delicate relationship between the compartmentalized modeling of infectious diseases and the next-generation approach. As we have seen in the above example, multiple compartmentalized models result in the same reproductive threshold.

### The Impact of the Asymptomatic Class on the Reproductive Threshold

The goal of this section is to investigate how the reproductive threshold arising from the $SEY AR$ model behaves in the case where asymptomatic carriers are not transmitting the disease. The natural parameter space of the $SEY AR$ model corresponding to the reproductive threshold is

$$\begin{aligned} \varTheta :=\left\{ (\varOmega _{{H}},\varOmega _{{M}},\xi _{{H}},\xi _{{M}},\beta _A,\beta _Y,\beta _{{M}},\gamma ,\tau ,\delta ,\sigma ,\lambda _{AR},\lambda _{YR},U_{_{{\textit{low}}}}) \in {\mathbb {R}}^{14}_{\tiny {>0}}\right\} , \end{aligned}$$

where ${\mathbb {R}}^n_{\tiny {>0}}:=\{x \in {\mathbb {R}}^n {:} x_i>0 \text {for} i=1,\ldots ,n\}$. The set $\varTheta$ consists of all possible positive, epidemiologically reasonable, parameter values for the $SEY AR$ model in which the reproductive threshold depends upon. Although the disease-induced death rate $\delta$ is allowed to be non-negative, the analysis presented in this section is unaffected by this minor detail. Under this formalization, neglecting the disease transmission and recovery rates of asymptomatic human hosts on the reproductive threshold formally corresponds to restricting the model to the following *A-nullified* parameter configuration space ${\tilde{\varTheta }}$, defined as follows:

$$\begin{aligned} {\tilde{\varTheta }}:=\left\{ (\varOmega _{{H}},\varOmega _{{M}},\xi _{{H}},\xi _{{M}},\beta _Y,\beta _{{M}},\gamma ,\tau ,\delta ,\sigma ,\lambda _{YR},U_{_{{\textit{low}}}}) \in {\mathbb {R}}^{12}_{\tiny {>0}}: \beta _A=\lambda _{AR}=0\right\} . \end{aligned}$$

Consider a typical element $\varTheta _0 \in \varTheta$ listed below:

$$\begin{aligned} \varTheta _0=\left( \varOmega _{H_0},\varOmega _{M_0},\xi _{H_0},\xi _{M_0},\beta _{A_0},\beta _{Y_0},\beta _{M_0},\gamma _{0},\tau _{0},\delta _0,\sigma _0,\lambda _{{AR}_0},\lambda _{{YR}_0},U_{{_{{\textit{low}}}}_{_0}}\right) . \end{aligned}$$

In a similar fashion, the dual element ${\tilde{\varTheta }}_0 \in {\tilde{\varTheta }}$ corresponding to the symptomatic class *Y* is given as follows:

$$\begin{aligned} {\tilde{\varTheta }}_0=\left( \varOmega _{H_0},\varOmega _{M_0},\xi _{H_0},\xi _{M_0},\beta _{Y_0},\beta _{M_0},\gamma _{0},\tau _{0},\delta _0,\sigma _0,\lambda _{{YR}_0},U_{{_{{\textit{low}}}}_{_0}}\right) . \end{aligned}$$

The dual element ${\tilde{\varTheta }}_0$ is comprised of the same fixed parameter configuration as described by $\varTheta _0$, but with the asymptomatic progression rates specified above set equal to zero. To emphasize the asymptotic dynamic influence of the asymptomatic class *A* on the reproductive threshold ${\mathcal {R}}_0$ (7) arising from the $SEY AR$ model (4), subject to a given fixed parameter configuration, the following notation is employed ${\mathcal {R}}_A:={\mathcal {R}}_0\Big |_{\varTheta _0}$. By denoting ${\mathcal {R}}_Y:={\mathcal {R}}_0\Big |_{{\tilde{\varTheta }}_0}$, the size relationship between the two quantities ${\mathcal {R}}_A$ and ${\mathcal {R}}_Y$ is captured below in the following theorem.

#### Theorem 4

(Impact of the asymptomatic class on the reproductive threshold) Let ${\mathcal {R}}_0$ be the threshold quantity (7) arising from Lemma 1 and consider the following fixed parameter configuration vectors $\varTheta _0 \in \varTheta$ and ${\tilde{\varTheta }}_0 \in {\tilde{\varTheta }}$, corresponding to the $SEY AR$ model (4), defined as above. Denote ${\mathcal {R}}_A:={\mathcal {R}}_0\Big |_{\varTheta _0}$ and ${\mathcal {R}}_Y:={\mathcal {R}}_0\Big |_{{\tilde{\varTheta }}_0}$, then it follows that ${\mathcal {R}}_Y<{\mathcal {R}}_A$.

This theorem means that ${\mathcal {R}}_0$ increases when there are asymptomatic individuals capable of transmitting the disease. The proof of the above theorem is located in “Proof of Theorem 4 (See Page 21)” of appendix. The above inequality is strict. Therefore, neglecting to account for asymptomatic carriers results in an underestimation of the reproductive threshold. To demonstrate the theoretical estimate provided in Theorem 4, the numerical values of ${\mathcal {R}}_A$ and ${\mathcal {R}}_Y$, along with the entomological inoculation rate (*EIR*) and parameter configuration space classifications, introduced via definition (1) in Sect. 3.2 are presented in Table 3 of Sect. 7.2. These numerical values correspond to the following three high transmission sites: Kaduna in Nigeria, Namawala in Tanzania, and Butelgut in Papua New Guinea. The parameter values associated with each site are listed in “Parameter Values” of appendix.

Previously, it was shown that ${\mathcal {R}}_0$ can be written in the following form:

9 $$\begin{aligned} {\mathcal {R}}_0=\sigma \prod _{i=1}^6 \sqrt{r_i}, \end{aligned}$$

where each term $\sqrt{r_i}$ is defined as in Sect. 3.2. Care needs to be taken when arbitrarily substituting zero parameter values into the reproductive threshold ${\mathcal {R}}_0$ (9). Clearly, if the human biting rate $\sigma =0$ or mosquito-to-human transmission probability $\beta _{{M}}=0$, then it follows that ${\mathcal {R}}_0=0$. These quantities effectively nullifying the reproductive threshold stand to epidemiological reason and correspond to the following scenarios, respectively: (1) no mosquitoes are biting humans, and (2) mosquitoes are not transmitting the disease. Furthermore, if both the asymptomatic $\beta _A$ and symptomatic $\beta _Y$ transmission probabilities are equal to zero, then the reproductive threshold will be identically zero, as infected humans are not transmitting the disease to susceptible mosquitoes.

However, it is implied that while one (or possibly all) of such parameter values may be equal to zero, the other parameter values in which the threshold depends on will be such that it is well defined. This problem is primarily due to the inclusion of vital dynamics for the human and mosquito populations into the dynamical system (4). For example, if one lets the human recruitment rate $\varOmega _{{H}}=0$ or the mosquito mortality rate $\xi _{{M}}=0$, then a singularity occurs and ${\mathcal {R}}_0$ ceases to be well defined. Although the mosquito mortality rate being equal to zero is unreasonable in an epidemiological sense, as all mosquitoes experience death, it is informative to study how this parameters size effects ${\mathcal {R}}_0$, as in general, it is desirable to increase such a parameter when introducing control measures into the system. This attests to the subtle fact that after including vital dynamics into a given compartmentalized infectious disease model, one cannot expect to obtain qualitative information about the reproductive threshold in the absence of vital dynamics by simply setting the corresponding terms equal to zero. To properly study the asymptotic behavior of such models without vital dynamics included, one would have to go back to the original model derivation and not include them from the beginning, then proceed to calculate the threshold arising from the modified system.

i Consider the following fixed parameter configuration vector $\varTheta _1\in {\mathbb {R}}^{13}_{\tiny {>0}}$ defined as follows:
  $$\begin{aligned} \varTheta _1:=\left( \varOmega _{H_0},\varOmega _{M_0},\xi _{H_0},\beta _{A_0},\beta _{Y_0},\beta _{M_0},\gamma _{0},\tau _{0},\delta _0,\sigma _0,\lambda _{{AR}_0},\lambda _{{YR}_0},U_{{_{{\textit{low}}}}_{_0}}\right) . \end{aligned}$$
  Then, by Eq. (9) and the fact that the square root function is uniformly continuous on $[0,+\infty )$ (so that the limit can be taken inside), it follows that
  $$\begin{aligned} \lim _{\xi _{{M}} \rightarrow +\infty } {\mathcal {R}}_0\Big |_{\varTheta _1}&=\sigma \sqrt{r^0_1r^0_3r^0_6}\cdot \sqrt{\lim _{\xi _{{M}} \rightarrow +\infty }\frac{\tau _{0}\xi _{H_0}\beta _{M_0}}{\xi ^2_{{M}}\left( \tau _{0} +\xi _{{M}} \right) }}, \\&=\sigma \sqrt{r^0_1r^0_3r^0_6} \cdot 0,\\&=0,\\ \end{aligned}$$
  where the superscripts appearing above each appropriate $i\text {th}$ term denote the fact that these quantities are fixed and thus invariant under the limit operation.
  There exists a natural number $\xi _{M_{n_0}}\in {\mathbb {N}}$ such that for all $\xi _{M_n}\ge \xi _{M_{n_0}}$, it follows that ${\mathcal {R}}_0 \in [0,1)$. This qualitative observation can be interpreted as follows: Provided a scenario such that all of the associated model parameters are fixed epidemiologically reasonable quantities, if control measures sufficient to increase the vector death rate are introduced into the model, then the corresponding reproductive threshold arising from the model will be lowered and eventually fall below unity. This is consistent with the discussion in Sect. 3.2.
ii In a similar fashion, consider the following fixed vectors $\left( \varTheta _2, \varTheta _3\right) \in {\mathbb {R}}^{13}_{\tiny {>0}} \times {\mathbb {R}}^{13}_{\tiny {>0}}$, defined as follows:
  $$\begin{aligned} \varTheta _2:=\left( \varOmega _{H_0},\xi _{H_0},\xi _{M_0},\beta _{A_0},\beta _{Y_0},\beta _{M_0},\gamma _{0},\tau _{0},\delta _0,\sigma _0,\lambda _{{AR}_0},\lambda _{{YR}_0},U_{{_{{\textit{low}}}}_{_0}}\right) \end{aligned}$$
  and
  $$\begin{aligned} \varTheta _3:=\left( \varOmega _{M_0},\xi _{H_0},\xi _{M_0},\beta _{A_0},\beta _{Y_0},\beta _{M_0},\gamma _{0},\tau _{0},\delta _0,\sigma _0,\lambda _{{AR}_0},\lambda _{{YR}_0},U_{{_{{\textit{low}}}}_{_0}}\right) . \end{aligned}$$
  Then, it follows that
  $$\begin{aligned} \lim _{\varOmega _{{M}} \rightarrow +\infty } {\mathcal {R}}_0\Big |_{\varTheta _2}&=\sigma \prod _{i=2}^6 \sqrt{r^0_i}\cdot \sqrt{\lim _{\varOmega _{{M}} \rightarrow +\infty }\frac{\varOmega _{{M}}}{\varOmega _{H_0}}}, \\&=+\infty ,\\ \end{aligned}$$
  and
  $$\begin{aligned} \lim _{\varOmega _{{H}} \rightarrow 0^+} {\mathcal {R}}_0\Big |_{\varTheta _3}&=\sigma \prod _{i=2}^6 \sqrt{r^0_i}\cdot \sqrt{\lim _{\varOmega _{{H}} \rightarrow 0^+}\frac{\varOmega _{M_0}}{\varOmega _{{H}}}}, \\&=+\infty .\\ \end{aligned}$$
  The above analysis demonstrates how ${\mathcal {R}}_0$ behaves with respect to the human and mosquito recruitment rates. As we will see in Sect. 7.1, if a given population, e.g., the three sites from which the parameter values are taken for this work, has a relatively small human recruitment rate and relatively large mosquito recruitment rate, then the factor $r_1$ will dramatically contribute to the size of ${\mathcal {R}}_0$. For example, in the case of the Kaduna site $r_1\approx 14{,}762{,}941.18$. The fractional multipliers $r_i$ for $i=2,3,4$ will reduce the resulting size of this quantity, as they are all strictly less than unity. Further reduction in the size of the remaining quantity in the decomposition of ${\mathcal {R}}_0$ depends on the terms $\sigma$, $r_5$, and $r_6$. These remaining factors will lower the resulting threshold provided that the mosquitoes are biting a relatively small amount of humans per unit time and transmitting less than they are dying off. Additionally, the asymptomatic and symptomatic human hosts need to be transmitting at a sufficiently low rate. For this reason, we must introduce control measures which both reduce the various disease transmission probabilities involved and increase the vector death rate.

Setting the parameters $\tau$ and $\gamma$ equal to zero obviously results in ${\mathcal {R}}_0=0$. However, these scenarios are not considered as the mosquito and human latent periods $\tau$ and $\gamma$ are considered to be intrinsic properties of the vector and host, respectively. Moreover, these specific terms only appear in the factors $\sqrt{r_i}$ for $i=2,3$ which are strictly bounded above by unity. Additionally, we do not consider the bizarre cases that the human mortality rate $\xi _{{H}}=0$ or mosquito recruitment rate $\varOmega _{{M}}=0$. Although both cases result in effectively nullifying the threshold quantity ${\mathcal {R}}_0$, humans are not immortal and the mosquito recruitment rate is usually relatively large. Letting this particular parameter value be equal to zero would imply the epidemiologically unreasonable scenario that there are no mosquitoes present in the region being considered. Neither do we consider the qualitative behavior of ${\mathcal {R}}_0$ if $\xi _{{H}}$ is sufficiently large, as introducing a human transmission-blocking control measure which also increases the human death rate of a given population is not an ethical control method. It is important to note that emphasis is made on the terms which are related to utilized control measures, i.e., the measures which have an effect on the mosquito death rate and the various transmission probabilities involved. Control measures will be formally introduced and covered in Sect. 6.

## Endemic Equilibria and Bifurcation Analysis

An endemic equilibrium occurs when disease persists in the population. For this reason, endemic equilibrium (EE) points are equilibrium points where some of the state variables corresponding to the infected classes are positive. Most epidemic models exhibit a dichotomy in terms of bifurcations that occur at the threshold ${\mathcal {R}}_0=1$, namely super-critical (forward) and sub-critical (backward). These have drastically different epidemiological implications. A forward bifurcation happens when ${\mathcal {R}}_0$ crosses unity from below and, as a result, a small positive asymptotically stable super-threshold equilibrium appears and the disease-free equilibrium losses its stability. Backward bifurcation happens when ${\mathcal {R}}_0<1$ and a small positive unstable sub-threshold equilibrium appears, while the disease-free equilibrium and a larger positive equilibrium are locally asymptotically stable.

From an epidemiological viewpoint, a forward bifurcation is more desirable as it results in the reproductive number being below unity to be sufficient to ensure that an epidemic does not occur. In the presence of a backward bifurcation, the reproductive number being below unity is no longer sufficient, as sub-threshold endemic equilibria can arise in response to perturbations of specific parameters.

Due to the presence of the term $e^{-\varepsilon }=\sum _{n=0}^\infty \frac{(-1)^n}{n!}\left( \frac{E}{N_{{H}}}\right) ^n$, one cannot obtain a closed-form expression for the endemic equilibrium solutions of system (4). However, we turn to a variant of the center manifold theorem, introduced by Castillo-Chavez and Song (2004), to show the existence of non-trivial equilibrium solutions of the $SEY AR$ model (4) near the DFE. This section is focused on the nonlinear stability analysis corresponding to the $SEY AR$ model. More precisely, the following theorem concerning its bifurcation behavior is proven.

### Theorem 5

(Bifurcation analysis for the $SEY AR$ model) Let ${\mathcal {R}}_0=1$ and the positive quantities $\eta _1$ and $\eta _2$ be defined as follows:

$$\begin{aligned} \eta _1&:=\, Z_6 Q_2 + \frac{\tau ^2 Z_1 Q^2_1 Q^2_4}{K_1 \xi _{{M}}}\left( 1+ \frac{K_2}{K_4} + \frac{K_3}{K_5}+ Q_0\right) \\&\qquad +\,\frac{\tau Z_2 K_2}{K_4 K_7}\left( \frac{\lambda _{RS} Q_0}{K_6} + 1 + Q_0\right) +\frac{\tau Z_3 K_3}{K_5 K_7}\left( \frac{\lambda _{RS} Q_0}{K_6} + 1 + Q_0\right) \\&\qquad +\,\frac{\tau Z_7 Q_4 Q_1 K_2}{\xi _{{M}} K_4}+\frac{\tau Z_5 Q_4 Q_1 K_3}{\xi _{{M}} K_5} + \frac{2\tau Z_2 K^2_2}{K^2_4 K_7}+ \frac{2\tau Z_3 K^2_3}{K^2_5 K_7},\\ \eta _2&:= \, \frac{\tau Z_2 K_1 K_2}{K_4 K_6 K_7}+\frac{\tau Z_3 K_1 K_3}{K_5 K_6 K_7}+\frac{\tau Z_4 K_2 K_3}{K_4 K_5 K_7} + Z_6 Q_3,\\ \end{aligned}$$

where the terms labeled $K_i$, $Q_i$, and $Z_i$, for $1=1,\ldots ,7$, are defined in “Appendix”. If the parameter $\varLambda$ is defined as

10 $$\begin{aligned} \varLambda :=\frac{\eta _2}{\eta _1}, \end{aligned}$$

then the $SEY AR$ model (4) exhibits a sub-critical bifurcation, provided that $\varLambda >1$, and super-critical bifurcation, provided $\varLambda <1$.

The formal proof of the above theorem can be found in “Proof of Theorem 5 (See Page 25)” of appendix. Moreover, a verification of the entries of the Jacobian and Hessian evaluated at the DFE is provided in the electronic supplementary material. As previously mentioned, Theorem 5 is proven by making use of an application of the center manifold theorem (Castillo-Chavez and Song 2004), adapted to the case of nonlinear dynamical systems. As in the case of most malaria models appearing throughout scientific literature, the type of bifurcation experienced by the system is completely determined by the sign of the *a*-term (18), listed in the appendix as Theorem 7. In the case of the $SEY AR$ model (4) $a \propto (\eta _2-\eta _1)$, resulting in a size constraint on $\varLambda$. In an epidemiological setting, it is desirable for the bifurcation, if it exists, to be super-critical, i.e., forward. Theorem 5 tells us that if one wants to avoid the case of a sub-critical bifurcation from occurring, we must demand the quantity $\varLambda <1$ in addition to ${\mathcal {R}}_0<1$.

## Incorporating Control Measures into the $SEY AR$ Model

The entomological inoculation rate (EIR) is a meaningful epidemiologic predictor that serves as a good measure of malaria intensity in a given region (Killeen et al. 2000). In 2007, Smith et al. estimated the reproductive number for 121 African populations. These estimates can be found in Figure 2 (Smith et al. 2007, p. 0534), where two numerical plots are displayed in which the reproductive number estimates are compared with the entomological inoculation rate of the populations under consideration. One plot corresponds to heterogeneous biting and transmission-blocking immunity taken into account in the parameter estimates and the other without. In both cases, the quantities were shown to be directly proportional, that is, regions with a relatively large (small) EIR also have a relatively large (small) reproductive number. In other words, regions with relatively large EIR values of each region also have relatively large ${\mathcal {R}}_0$ values. In areas with large ${\mathcal {R}}_0$, it is unlikely that one single control measure will be sufficient to stop the disease expansion (Smith et al. 2007).

In practice, vaccine-conveyed immunity is not one hundred percent effective. This fact is accounted for by the vaccine efficacy $V_f$, which denotes the percentage of protection each vaccinated individual has. If $V_p$ denotes the proportion of the population that has been vaccinated, i.e., the vaccine coverage, the product $V_f V_p$ stands for the fraction of the population under consideration that is protected, so that the remaining proportion $(1-V_f V_p)$ is not directly protected, with respect to vaccine-conveyed immunity. As a result, vaccination controls are incorporated into the model by defining the weight ${\bar{v}}:=(1-V_f V_p)$. Therefore, the control-modified progression rates are given by the following equations:

$$\begin{aligned} {\left\{ \begin{array}{ll} {\tilde{\lambda }}_{EA} &{}:={\bar{v}}\gamma u(\varepsilon ),\\ {\tilde{\lambda }}_{EY} &{}:={\bar{v}}\gamma (1-u(\varepsilon )).\\ \end{array}\right. } \end{aligned}$$

Insecticide-treated nets (ITNs) are the most prominent malaria preventive measure for large-scale deployment in highly endemic areas such as sub-Saharan Africa (Lengeler 2004). ITNs are nets coated with synthetic pyrethroid insecticides. Many studies have shown them to both kill and repel mosquitoes. In a recent study, a regression analysis of the protective efficacy on the transmission intensity, as measured by the EIR, was performed at the following four different endemic regions of Africa: Burkina Faso, The Gambia, Ghana, and Kenya. It was noted that the protective efficacy was lower in areas with a higher EIR, which was consistent with the original hypothesis that relative impact is lower in areas with higher entomological inoculation rates (Lengeler 2004). Moreover, in the case of homogeneous biting, $99.95\%$ ITN coverage was predicted to be necessary (Smith et al. 2007).

Regarding the ITN coverage, let the symbol $\rho _f$ denote the protective efficacy, i.e., the percentage reduction in malaria episodes due to bed net usage. Upon letting $\rho _p$ be the proportion of ITN usage, i.e., the percentage decrease in transmission due to the employment of ITNs, then the reduction in mosquito-to-human transmission is captured by the multiplier $(1-\rho _f\rho _p)$. Additionally, let $\xi _{_{{\text {ITN}}}}$ denote the maximum ITN-induced death rate for the mosquito population. Following (Agusto et al. 2013), it is assumed that ITN usage reduces the effective human-to-mosquito effective contact probabilities $\beta _A$ and $\beta _Y$ and increases the mosquito mortality rate $\xi _{{M}}$. Thus, the effects of ITN usage on the disease transmission dynamics of the $SEY AR$ model (4) are accounted for by the following modification:

$$\begin{aligned} {\left\{ \begin{array}{ll} {\tilde{\beta }}_{A} &{}:=(1-\rho _f\rho _p)\beta _A,\\ {\tilde{\beta }}_{Y} &{}:=(1-\rho _f\rho _p)\beta _Y,\\ {\tilde{\xi }}_{{M}} &{}:=\xi _{{M}} + \rho _f\rho _p \xi _{_{{\text {ITN}}}}.\\ \end{array}\right. } \end{aligned}$$

Therefore, the resulting control-modified variant of the $SEY AR$ model (4) expanded in the original model parameters is

$$\begin{aligned} {\left\{ \begin{array}{ll} {\dot{S}} =\varOmega _{{H}} + \lambda _{RS} R - \left( \sigma \beta _{{M}} \frac{M_{{I}}}{N_{{H}}} + \xi _{{H}} \right) S, \\ {\dot{E}} =\sigma \beta _{{M}} \frac{M_{{I}}}{N_{{H}}}S - (\gamma +\xi _{{H}})E, \\ {\dot{Y}} =(1-V_f V_p)\gamma (1-u(\varepsilon )) E - \left( \xi _{{H}} +\delta + \lambda _{YR}\right) Y, \\ {\dot{A}} =(1-V_f V_p)\gamma u(\varepsilon ) E - \left( \lambda _{AR}+\xi _{{H}}\right) A, \\ {\dot{R}} =\lambda _{AR}A + \lambda _{YR}Y - \left( \lambda _{RS} +\xi _{{H}}\right) R, \\ \dot{M_{{S}}} =\varOmega _{{M}} - \left( \xi _{{M}} + \rho _f\rho _p \xi _{_{{\text {ITN}}}} + \sigma (1-\rho _f\rho _p)\beta _Y \frac{Y}{N_{{H}}} +\sigma (1-\rho _f\rho _p)\beta _A \frac{A}{N_{{H}}} \right) M_{{S}}, \\ \dot{M_{{E}}} =\sigma (1-\rho _f\rho _p)\left( \beta _Y \frac{Y}{N_{{H}}} +\beta _A \frac{A}{N_{{H}}}\right) M_{{S}} - \left( \xi _{{M}} + \rho _f\rho _p \xi _{_{{\text {ITN}}}} + \tau \right) M_{{E}}, \\ \dot{M_{{I}}} =\tau M_{{E}}- (\xi _{{M}} + \rho _f\rho _p\xi _{_{{\text {ITN}}}}) M_{{I}}. \end{array}\right. } \end{aligned}$$

Assuming that the initial exposed proportion is zero, i.e., $\varepsilon _0=0$, the corresponding vaccination control-modified reproductive threshold ${\mathcal {R}}^{{\bar{v}}}_0$ is given by the following formula:

11 $$\begin{aligned} {\mathcal {R}}^{{\bar{v}}}_0 =&\, \sqrt{\frac{\sigma ^2 \tau \gamma \varOmega _{{M}} \xi _{{H}} \beta _{{M}}(1-\rho _f\rho _p){\bar{v}}}{(\xi _{{M}} + \rho _f\rho _p \xi _{_{{\text {ITN}}}})^2(\gamma + \xi _{{H}})(\tau + \xi _{{M}} + \rho _f\rho _p \xi _{_{{\text {ITN}}}})\varOmega _{{H}}} } \nonumber \\&\, \times \sqrt{\left( \frac{\beta _Au_{_{{\textit{low}}}}}{\lambda _{AR} + \xi _{{H}}} - \frac{\beta _Y(u_{_{{\textit{low}}}}-1)}{\lambda _{YR} + \xi _{{H}} + \delta } \right) }. \end{aligned}$$

## Sensitivity Analysis and Numerical Results

### Sensitivity Analysis

Uncertainty is usually present in data collection and presumed parameter values. In this section, a sensitivity analysis is applied to classify the parameters which have the highest impact on the reproductive threshold ${\mathcal {R}}_0$. This provides a way to determine which parameter values should be targeted by intervention strategies. A parameter with a relatively large sensitivity index should be estimated with precision, while a parameter with a relatively small sensitivity index does not require as much effort.

Let $\varTheta$ be defined as in Sect. 4.3, then it is of trivial consequence that ${\mathcal {R}}_0 \in C^1(\varTheta )$. Due to this fact and that we have an explicit expression for ${\mathcal {R}}_0$ of the $SEY AR$ model (4), we arrive at the following definition.

#### Definition 2

(*Sensitivity index of the reproductive threshold* (Chitnis et al. 2008)) Consider the reproductive threshold ${\mathcal {R}}_0 \in C^1(\varTheta )$ given by Eq. (7) in Sect. 3.2, and let $\{e_i {:} 1\le i \le 14\}$ be the canonical basis in ${\mathbb {R}}^{14}$. For ${\tilde{\rho }} \in \varTheta$ define $\rho _i :=\langle e_i,{\tilde{\rho }}\rangle$, where $\langle \cdot ,\cdot \rangle$ denotes the inner product in 14-dimensional Euclidean space, then the normalized forward sensitivity index of ${\mathcal {R}}_0$ with respect to the parameter $\rho _i$ is defined by the following differential equation:

$$\begin{aligned} \varUpsilon ^{{\mathcal {R}}_0}_{\rho _i}:=\frac{\partial {\mathcal {R}}_0}{\partial \rho _i} \times \frac{\rho _i}{{\mathcal {R}}_0}. \end{aligned}$$

The normalized forward sensitivity index provides a way to quantify the relative change in the given expression when the parameter changes. The sensitivity index is well defined, provided that ${\mathcal {R}}_0$ is at least in $C^1$ with respect to each parameter $\rho _i$.

The analytic formulas for the sensitivity indices are complex and do not offer much qualitative insight; as a result, we evaluate the indices at the parameter values corresponding to each location. Table 2 contains the sensitivity indices of ${\mathcal {R}}_0$ for the $SEY AR$ model (4) evaluated at the parameter values given in “Parameter Values” of appendix. The parameters are ordered from the most sensitive to the least.

**Table 2:** Sensitivity indices of ${\mathcal {R}}_0$

Parameter	Kaduna	Namawala	Butelgut
$\xi _{{M}}$	$-$ 1.27500	$-$ 1.250000	$-$ 1.291666
$\sigma$	$+$ 1.00000	$+$ 1.000000	$+$ 1.000000
$\varOmega _{{M}}$	$+$ 0.50000	$+$ 0.500000	$+$ 0.500000
$\beta _{{M}}$	$+$ 0.50000	$+$ 0.500000	$+$ 0.500000
$\varOmega _{{H}}$	$-$ 0.49999	$-$ 0.500000	$-$ 0.499999
$\beta _Y$	$+$ 0.49369	$+$ 0.493906	$+$ 0.493975
$U_{_{{\textit{low}}}}$	$-$ 0.48739	$-$ 0.487812	$-$ 0.487951
$\lambda _{YR}$	$-$ 0.37495	$-$ 0.389920	$-$ 0.395175
$\xi _{{H}}$	$+$ 0.30742	$+$ 0.332370	$+$ 0.341059
$\tau$	$+$ 0.27499	$+$ 0.250000	$+$ 0.291666
$\gamma$	$+$ 0.07364	$+$ 0.063490	$+$ 0.060000
$\beta _A$	$+$ 0.00630	$+$ 0.006093	$+$ 0.006024
$\lambda _{AR}$	$-$ 0.00610	$-$ 0.005935	$-$ 0.005877
$\delta$	$-$ 0.00001	$-$ 0.000007	$-$ 0.000006

This table contains the sensitivity indices of ${\mathcal {R}}_0$ evaluated at Kaduna, Namawala, and Butelgut

A verification of the numerical entries in Table 2 is provided in the electronic supplementary material. The sensitivity analysis conducted on the reproductive threshold of the model above shows that the most sensitive parameters are the mosquito mortality rate $\xi _{{M}}$ and the human biting rate $\sigma$. Conversely, the least sensitive is the disease-induced death rate for humans $\delta$. The sensitivity indices listed in the above tables can be viewed as growth measurements of the reproductive threshold with respect to the parameter under consideration. Furthermore, the result of the sensitivity analysis shows that mosquito parameters are most important, which is consistent with the classic model of Ross–Macdonald; however, the magnitude of the sensitivity indices varies due to the reconfiguration of the compartments in our model, and the addition of new parameters.

Without loss of generality, attention is turned to the Kaduna location. Concerning Kaduna, an increase in $\xi _{{M}}$ by $10\%$ will result in a decrease in ${\mathcal {R}}_0$ by $12.75\%$. Similarly, an increase in $\sigma$ by $10\%$ will cause a $10\%$ increase in ${\mathcal {R}}_0$. Also, it is worth noting the asymptomatic recovery and effective contact probabilities $\lambda _{AR}$ and $\beta _A$ are relatively less sensitive than the symptomatic human-related terms $\lambda _{YR}$ and $\beta _Y$. Furthermore, an increase in $\varOmega _{{M}}$ by $10\%$ results in an increase of ${\mathcal {R}}_0$ by approximately $5\%$, and a $10\%$ decrease in $\varOmega _{{H}}$ will cause a $5\%$ increase in ${\mathcal {R}}_0$. These point-wise numerical observations are consistent with the qualitative analysis presented in Sect. 4.3.

The hierarchy of sensitivity that the $SEY AR$ model parameters obey is common among many compartmentalized homogeneous population malaria models appearing in the literature, e.g., Chitnis et al. (2008) and Roop-O et al. (2015). The two most sensitive parameters correspond to the vector population. These parameters have the property that one is directly proportional to the reproductive number, while the other is inversely proportional.

Increasing the mosquito death rate will also reduce the human biting rate, as the average mosquito life span is shortened. This is beneficial from a practical standpoint, as the parameter which is desirable to increase has the additional effect of decreasing the parameter that is desirable to decrease. Theoretically, this aids in the designing of programs for disease control, as it isolates the parameters that should be targeted for reduction by intervention strategies. Insecticide-treated bed nets and indoor residual spraying are among the most common methods used for such purposes. In Sect. 6, these control measures are incorporated into the model.

### Numerical Results

Displayed below is a table containing numerical values for the following:

(i) the parameter configuration space classification $C_1-C_2$, introduced via Definition 1 in Sect. 3.2,
(ii) the EIR corresponding to each location,
(iii) the reproductive threshold accounting for asymptomatic carriers ${\mathcal {R}}_0$, given by Eq. (7),
(iv) the reproductive threshold neglecting asymptomatic carriers ${\mathcal {R}}_Y$, as discussed in Sect. 4.3.

These numerical values correspond to the following three high transmission sites: Kaduna in Nigeria, Namawala in Tanzania, and Butelgut in Papua New Guinea. The parameter values associated with each site are listed in “Parameter Values” of appendix.

**Table 3:** EIR and threshold quantities

Site	Classification	EIR	${\mathcal {R}}_0$	${\mathcal {R}}_Y$
Kaduna	$A {\textit{-dominant}}{/}-6.00^{\mathrm{a}}$	$120^{\mathrm{b}}$	$393.05^{\mathrm{e}}$	$390.56^{\mathrm{f}}$
Namawala	$A {\textit{-dominant}}{/}-6.24^{\mathrm{a}}$	$329^{\mathrm{c}}$	$68.38^{\mathrm{e}}$	$67.96^{\mathrm{f}}$
Butelgut	$A {\textit{-dominant}}{/}-6.32^{\mathrm{a}}$	$517^{\mathrm{d}}$	$81.12^{\mathrm{e}}$	$80.63^{\mathrm{f}}$

$^{\mathrm{a}}$ These quantities are calculated from Definition 1 in Sect. 3.2

$^{\mathrm{b}}$ This value was taken from Service (1965)

$^{\mathrm{c}}$ This value was taken from Smith et al. (1993)

$^{\mathrm{d}}$ This value was calculated by Killeen et al. (2000), using data obtained from the following sources (Burkot et al. 1988; Graves et al. 1990)

$^{\mathrm{e}}$ These quantities were calculated from Eq. (7) assuming that the initial exposed proportion is zero, i.e., $\varepsilon _0=0$

$^{\mathrm{f}}$ These quantities were calculated by setting the asymptomatic progression rates equal to zero

A verification of the numerical threshold quantities presented in Table 3 is provided in the electronic supplementary material. One should observe that the sizes of the reproductive thresholds for the three sites under consideration are consistent with the sizes of the corresponding entomological inoculation rate values. Regions with a relatively large EIR value also have relatively large ${\mathcal {R}}_0$ values. As mentioned in Sect. 6, in areas with large reproductive thresholds, it is unlikely that one single control measure will be sufficient to stop the disease expansion.

Additionally, the threshold quantities ${\mathcal {R}}_0={\mathcal {R}}_A$ and ${\mathcal {R}}_Y$ obey the theoretical estimate provided in Theorem 4. Therefore, neglecting to account for asymptomatic carriers results in an underestimation of the reproductive threshold corresponding to each location. The theoretical estimate provided in Theorem 4 holds for all possible positive epidemiologically reasonable quantities. Furthermore, the numerical entries displayed in Table 2 of Sect. 7.1 and those listed in Table 3 are point-wise evaluations.

There have been reports which indicate that asymptomatic carriers in a given population may transmit the disease at a higher rate than the symptomatic (Vallejo et al. 2016). In this case, the sensitivity hierarchy will possess a different ordering and the size differences in the threshold quantities displayed above will be larger.

## Conclusions and Discussions

This study clearly shows that the existence of asymptomatic individuals results in a strict underestimation of ${\mathcal {R}}_0$ and provides the means to quantify this influence. It also provides the means to study NAI as the factor that drives asymptomaticity. As mentioned by Doolan et al. (2009), the exploration of NAI is key to the rational development and deployment of vaccines and other malaria control methods corresponding to any given population at risk. Therefore, it is a necessary foundation upon to build strategies of eradication by any means.

The $SEY AR$ model (4) accounts for the impact that the exposure-dependent naturally acquired immune proportion has on asymptomatic carriers and malaria disease transmission dynamics. Through making use of the IVP (1), the infected compartment *I* is effectively decomposed into two mutually disjoint sub-compartments accounting for symptomatic and asymptomatic individuals. This results in a model which does not fall into a sub-class of the type studied by Filipe et al. (2007).

Current asymptomatic models appearing in the literature are formed by inserting a state-invariant constant control parameter or a sum of transcendental expressions involving various state-invariant immunity acquisition rates. The $SEY AR$ model is derived by a separation through means of the NAI proportion of a population which depends on exposure through Eq. (2). After deriving the model and addressing the issues of well-posedness and stability analysis, a nonlinear stability analysis is performed in which the bifurcation behavior of the model is characterized. A sensitivity analysis is carried out, and generalized control measures are introduced in the model. Moreover, numerical values of various quantities discussed throughout this work are provided for the following three high transmission sites: Kaduna in Nigeria, Namawala in Tanzania, and Butelgut in Papua New Guinea. A brief summary of highlights drawn from the conclusions of this work is presented in the form of a list below:

1. In Sect. 3.1, it was shown that the $SEY AR$ model (4) is mathematically and epidemiologically well posed, provided the initial data satisfied suitable regularity assumptions. Additionally, in Theorem 3, a mathematically precise and epidemiologically reasonable lower bound for the feasible region of the model was provided.
2. In Lemma 1 of Sect. 3.2, the $SEY AR$ model (4) was shown to satisfy the threshold condition. Moreover, ${\mathcal {R}}_0$ was decomposed into a product to properly analyze the size contribution of each factor involved. Provided that the mosquito mortality rate $\xi _{{M}}$ can be made large enough so that the term $C_0$ compromised of fractional multipliers is sufficiently small, i.e., $C_0<1$, then the size of ${\mathcal {R}}_0$ is completely determined by the human-dependent factor $r_6$, defined in Sect. 3.2, which consists of a weighted difference of vital dynamics with the NAI proportion, recovery, and death rates. Motivated by the monotonic behavior of ${\mathcal {R}}_0$, a formal characterization of the parameter configuration space was introduced via Definition 1.
3. In Sect. 4.1, a modification was made to the $SEY AR$ model which accounts for the case when sterilizing immunity via premunition is negligible, thus eliminating the need for an *R* compartment. Moreover, in Sect. 4.2, relapse rates are introduced into the model and the corresponding reproductive threshold is calculated. In Sect. 4.3, an estimate is provided which characterizes the impact that the asymptomatic class has on the reproductive threshold. More precisely, it was shown that neglecting asymptomatic carriers results in an underestimation of the threshold.
4. In Sect. 5 use is made of Theorem 7 to show the existence of non-trivial sub-threshold equilibrium solutions near the DFE. More precisely, it was shown that the bifurcation experienced by the $SEY AR$ model (4) is forward or backward depending on the size of an auxiliary threshold parameter $\varLambda$ defined by (10). As it is desirable for no endemic equilibrium states to arise while ${\mathcal {R}}_0<1$, we impose the additional requirement that $\varLambda <1$.
5. In Sect. 6, control measures are incorporated into the $SEY AR$ model (4).
6. In Sect. 7.1, a sensitivity analysis is conducted on the reproductive number of the model for parameter configurations corresponding to high transmission settings. Additionally, a table was provided which contains the sensitivity indices of ${\mathcal {R}}_0$ with respect to each parameter. An ordering of the parameters from the most sensitive to least revealed that the most sensitive parameters were the mosquito mortality rate $\xi _{{M}}$ and human biting rate $\sigma$. The least sensitive was the disease-induced human death rate $\delta$. Additionally, the asymptomatic recovery rate $\lambda _{AR}$ and the transmission probability $\beta _A$ were shown to be relatively less sensitive than the symptomatic human-related terms $\lambda _{YR}$ and $\beta _Y$.
7. In Sect. 7.2, numerical results corresponding to the three high transmission sites mentioned above are provided. The numerical values of the threshold quantities were shown to be consistent with the theory presented in Sect. 4.3. As the sites of interest are high transmission areas with relatively high entomological inoculation rates, the reproductive thresholds were shown to be comparably large.

As directions of future research, it will be interesting to apply the method of decomposing the infected compartment by means of the related rate IVP (1) in Sect. 2 to other infectious diseases where asymptomatic individuals play a fundamental role in the disease dynamics. Additionally, it will be informative to consider extensions of the $SEY AR$ model formed by incorporating the other kinds of immunity mentioned in the introduction. Furthermore, it will be beneficial to introduce additional control measures into the models such as aerial fogging and a time-dependent treatment rate of symptomatic carriers. An important direction for future exploration is the study of *P. vivax* and *P. falciparum* co-infection.

In conclusion, the $SEY AR$ model (4) has provided us with a precise mathematical understanding of the relationship between the exposure-dependent nature of NAI and asymptomatic malaria disease transmission dynamics.

### Electronic supplementary material

Below is the link to the electronic supplementary material.

Supplementary material 1 (mw 35 KB)

Supplementary material 2 (mw 41 KB)

Supplementary material 3 (mw 33 KB)

Supplementary material 4 (mw 44 KB)

## Appendix

This section of the appendix contains formal proofs of the lemmas and theorems presented in Sect. 3. Listed below is an overview of the notation and mathematical framework utilized in the proofs. For an in-depth look into the function classes utilized in this work, and many other closely related topics in the field of harmonic analysis, the reader is referred to Fabec and Ólafsson (2014) and Grafakos (2008).

### Notation and Mathematical Framework

Firstly, let ${\mathbb {R}}_+:=\{x \in {\mathbb {R}} {:} x\ge 0\}$ be the space of nonnegative real numbers and *dt* denote the Lebesgue measure for any complex-valued measurable function $\varphi$. The $L^1({\mathbb {R}}_+)$ norm of $\varphi$ is defined as

$$\begin{aligned} \Vert \varphi \Vert _1:= \int _0^\infty | \varphi | \mathrm{d}t. \end{aligned}$$

Extensive use is made of the space of essentially bounded functions $L^\infty ({\mathbb {R}}_+)$ characterized by the following norm:

Analogous to the case of the essential supremum, the essential infimum is defined as

Some of the proofs require at least a bounded first derivative; however, a general $\varphi \in C^1({\mathbb {R}}_+)$ is not bounded. Due to this fact, we need to restrict our analysis to a smaller space possessing a higher degree of regularity. The most natural space to turn to is the sub-space denoted $C^1_b({\mathbb {R}}_+) \subset C^1({\mathbb {R}}_+)$. This is the Banach space of bounded continuous functions whose first derivative is also bounded, endowed with the norm

$$\begin{aligned} C^1_b({\mathbb {R}}_+):=\{\varphi \in C^1({\mathbb {R}}_+) {:} \Vert \varphi \Vert _{C^1_b} :=\Vert \varphi \Vert _\infty +\Vert {\dot{\varphi }}\Vert _\infty <+\infty \}. \end{aligned}$$

The convolution of two functions is defined as $\varphi _1 *\varphi _2(t)= \int _{0}^t \varphi _1(\tau )\varphi _2(t-\tau )d\tau$. Whenever $\varphi _1 *\varphi _2(t) \in L^1({\mathbb {R}}_+)$, the integral is finite and, as a result, well defined.

### Proofs

#### Proof of Theorem 1 (See Page 8)

##### Proof

The continuous composition of functions $u(t)=(u \circ \varepsilon )(t)$ solves the IVP (1) for all $\varepsilon \in C^2({\mathbb {R}}_+)$ in the solution space. By the non-negativity of $\varepsilon$, if the initial data $\varepsilon _0$ are such that

$$\begin{aligned} e^{\varepsilon _0}(u_{_{{\textit{low}}}}\,-u_{_{{\textit{high}}}}) + u_{_{{\textit{high}}}}\ge 0, \end{aligned}$$

then the solution $u \in C^2({\mathbb {R}}_+)$ is non-negative for all $t \in {\mathbb {R}}_+$. The above inequality is satisfied, provided $\varepsilon _0 \le \vartheta$ for $\vartheta$ defined by Eq. (3) in Sect. 2. $\square$

#### Proof of Theorem 2 (See Page 11)

##### Proof

The functions under consideration represent bounded, continuous, smoothly varying, nonnegative biological quantities. Consequently, it is reasonable to assume that ${\mathbf {x}} \in C^1_b({\mathbb {R}}_+)$. This reasoning combined with the fact that the vector field $\varPhi \in C^1$ is locally Lipschitz implies the existence of a unique solution ${\mathbf {x}}$. Moreover, the solution ${\mathbf {x}}$ depends continuously on the initial data and model parameters and can be continued to a maximal time interval (Smale et al. 2003). $\square$

#### Proof of Theorem 3 (See Page 12)

##### Proof

The case for the mosquito population is trivial, viz.

$$\begin{aligned} \lim _{t \rightarrow +\infty }N_{{M}}(t) = \frac{\varOmega _{{M}}}{\xi _{{M}}}. \end{aligned}$$

For the human population, let $L_t=e^{\int _{0}^t\xi _{{H}} ds}=e^{\small {\xi _{{H}} t}}$, so that

$$\begin{aligned} \dot{(L_tN_{{H}})}&= L_t \varOmega _{{H}}-\delta L_t Y, \\ L_t N_{{H}}(t)&= N_{{H}}(0) + \int _{0}^t L_s\varOmega _{{H}} \mathrm{d}s- \delta \int _{0}^t L_s Y(s)\mathrm{d}s,\\&= N_{{H}}(0) + \varOmega _{{H}} \left( \frac{e^{\small {\xi _{{H}} t}}}{\xi _{{H}}} - \frac{1}{\xi _{{H}}} \right) - \delta \int _{0}^t e^{\small {\xi _{{H}}s}} Y(s)\mathrm{d}s.\\ \end{aligned}$$

Therefore, the explicit solution is given by

$$\begin{aligned} N_{{H}}(t)&= e^{\small {-\xi _{{H}} t}} N_{{H}}(0) + \frac{\varOmega _{{H}} }{\xi _{{H}}}\left( 1-e^{\small {-\xi _{{H}} t}}\right) - \delta \int _{0}^t e^{\small {-\xi _{{H}}(t-s)}} Y(s)\mathrm{d}s, \\ \end{aligned}$$

or in operator form

$$\begin{aligned} N_{{H}}(t)=L_{-t}N_{{H}}(0)+ \frac{\varOmega _{{H}} }{\xi _{{H}}}\left( 1-L_{-t}\right) -\delta L_{-t} *Y(t). \end{aligned}$$

After passing to the limit, it follows that

12 $$\begin{aligned} \lim _{t \rightarrow +\infty }N_{{H}}(t) = \frac{\varOmega _{{H}}}{\xi _{{H}}}-\delta \lim _{t \rightarrow +\infty }L_{-t} *Y(t). \end{aligned}$$

In the absence of symptomatic infection, i.e., $Y=0$, it follows that $L_{-t} *Y(t)=0$, so that

$$\begin{aligned} \lim _{t \rightarrow +\infty }N_{{H}}(t) = \frac{\varOmega _{{H}}}{\xi _{{H}}}. \end{aligned}$$

As expected, $N_{{H}}$ asymptotes to the equilibrium population density of the human population. If symptomatic infection occurs in the population, by Hölder’s inequality it follows that

$$\begin{aligned} L_{-t} *Y(t)&\le \Vert Y \Vert _\infty \Vert L_{-(t-s)}\Vert _1, \\&= \Vert Y \Vert _\infty \int _{0}^t e^{\small {-\xi _{{H}}(t-s)}}\mathrm{d}s, \\&=\Vert Y \Vert _\infty \left[ \frac{1}{\xi _{{H}}} -\frac{e^{\small {-\xi _{{H}} t}}}{\xi _{{H}}}\right] , \end{aligned}$$

where the right-hand side of the above inequality is finite due to the assumption that $Y \in C^2({\mathbb {R}}_+) \cap C^1_b({\mathbb {R}}_+) \subset L^\infty ({\mathbb {R}}_+)$. By letting $t\rightarrow +\infty$, we arrive at the following estimate for the forcing term

$$\begin{aligned} \lim _{t \rightarrow +\infty } L_{-t} *Y(t) \le \frac{\Vert Y \Vert _\infty }{\xi _{{H}}}. \end{aligned}$$

The above estimate in combination with (12) yields the following lower bound

$$\begin{aligned} \lim _{t \rightarrow +\infty }N_{{H}}(t) \ge \frac{\varOmega _{{H}}}{\xi _{{H}}}- \delta \frac{\Vert Y \Vert _\infty }{\xi _{{H}}}. \end{aligned}$$

Therefore,

$$\begin{aligned} \lim _{t \rightarrow +\infty }N_{{H}}(t) \in \left[ \frac{\varOmega _{{H}} - \delta \Vert Y \Vert _\infty }{\xi _{{H}}},\frac{\varOmega _{{H}}}{\xi _{{H}}} \right] . \end{aligned}$$

Due to the restriction $N_{{H}} \in {\mathbb {R}}_+$ for all $t \in {\mathbb {R}}_+$, it follows that

$$\begin{aligned} \lim _{t \rightarrow +\infty }N_{{H}}(t) \in \left[ \alpha ,\frac{\varOmega _{{H}}}{\xi _{{H}}} \right] , \end{aligned}$$

where $\alpha$ is defined in the statement of the theorem. Furthermore, for system (6) we have that

$$\begin{aligned} {\dot{N}}_{{H}} \le \varOmega _{{H}}-\xi _{{H}} N_{{H}} = \xi _{{H}} \left( \frac{\varOmega _{{H}}}{\xi _{{H}}} - N_{{H}}\right) . \end{aligned}$$

Combining the above observation with a similar argument for $N_{{M}}$ yields

13 $$\begin{aligned} {\left\{ \begin{array}{ll} {\dot{N}}_{{H}} \le 0,\quad \text {if} \quad N_{{H}} \ge \frac{\varOmega _{{H}}}{\xi _{{H}}},\\ {\dot{N}}_{{M}} \le 0,\quad \text {if} \quad N_{{M}} \ge \frac{\varOmega _{{M}}}{\xi _{{M}}}. \end{array}\right. } \end{aligned}$$

Inequalities (13) imply that if the solution leaves the region $\varGamma$, then its derivative will instantaneously become negative, forcing it back to $\varGamma$. Moreover, if $x_i(0)=0$ for any $1 \le i \le 8$ in system (5), then it directly follows that ${\dot{x}}_i \ge 0$. Therefore, all trajectories tend to $\varGamma$ and are forward invariant. Due to this fact, it is sufficient to study the dynamics of the system on the smaller compact sub-space $\varGamma$. $\square$

#### Proof of Lemma 1 (See Page 14)

##### Proof

Firstly, we order the compartments so that the first five correspond to infected individuals and denote ${\mathbf {w}}=\left( E,Y,A,M_{{E}},M_{{I}},R,S,M_{{S}}\right) ^T$. The corresponding DFE is

$$\begin{aligned} {\mathbf {w}}_{\small {{dfe}}}=\left( 0,0,0,0,0,0,\frac{\varOmega _{{H}}}{\xi _{{H}}},\frac{\varOmega _{{M}}}{\xi _{{M}}}\right) ^T. \end{aligned}$$

Following the next-generation method, system (4) is rewritten in the following form:

$$\begin{aligned} \dot{{\mathbf {w}}}=\varPhi \left( {\mathbf {w}}\right) ={\mathcal {F}}\left( {\mathbf {w}}\right) -{\mathcal {V}}\left( {\mathbf {w}}\right) , \end{aligned}$$

where ${\mathcal {F}}:=\left( {\mathcal {F}}_1,\dots ,{\mathcal {F}}_8\right) ^T$ and ${\mathcal {V}}:=\left( {\mathcal {V}}_1,\dots ,{\mathcal {V}}_8\right) ^T$, or more explicitly

$$\begin{aligned} \begin{pmatrix} {\dot{E}}\\ {\dot{Y}}\\ {\dot{A}}\\ {\dot{M}}_{{E}}\\ {\dot{M}}_{{I}}\\ {\dot{R}}\\ {\dot{S}}\\ {\dot{M}}_{{S}}\\ \end{pmatrix} = \begin{pmatrix} \sigma \beta _{{M}} \frac{M_{{I}}}{N_{{H}}}S \\ 0 \\ 0 \\ \frac{\sigma \left( \beta _Y Y + \beta _A A\right) }{N_{{H}}} M_{{S}}\\ 0 \\ 0 \\ 0 \\ 0 \\ \end{pmatrix} - \begin{pmatrix} (\gamma +\xi _{{H}})E\\ -\gamma \left( 1-u(\varepsilon )\right) E + \left( \xi _{{H}} +\delta + \lambda _{YR}\right) Y\\ -\gamma u(\varepsilon ) E + \left( \lambda _{AR}+\xi _{{H}}\right) A \\ \left( \xi _{{M}} + \tau \right) M_{{E}}\\ -\tau M_{{E}} + \xi _{{M}} M_{{I}}\\ -\lambda _{AR}A - \lambda _{YR}Y +\left( \lambda _{RS} +\xi _{{H}}\right) R \\ -\varOmega _{{H}} - \lambda _{RS} R +\left( \sigma \beta _{{M}} \frac{M_{{I}}}{N_{{H}}} + \xi _{{H}} \right) S\\ -\varOmega _{{M}} + \left( \xi _{{M}} + \frac{\sigma \left( \beta _Y Y + \beta _A A\right) }{N_{{H}}} \right) M_{{S}} \\ \end{pmatrix}. \end{aligned}$$

In addition, the matrix ${\mathcal {V}}$ admits the decomposition ${\mathcal {V}}={\mathcal {V}}^{-}-{\mathcal {V}}^{+}$, where the component-wise definition is inherited. Biologically speaking: ${\mathcal {F}}_i$ is the rate of appearance of new infections in compartment *i*, ${\mathcal {V}}^{+}_i$ stands for the rate of transfer of individuals into compartment *i* by any other means and ${\mathcal {V}}^{-}_i$ is the rate of transfer of individuals out of compartment *i*. It is easy to see that ${\mathcal {F}},{\mathcal {V}}^{-},{\mathcal {V}}^{+}$ satisfy assumptions (i)–(v) in Theorem 6. As mentioned in the beginning of Sect. 3.1, to study the stability of the equilibrium points, we assume that each of the above vector fields is at least twice continuously differentiable. Define $U_{_{{\textit{low}}}}:=e^{\varepsilon _0}(u_{_{{\textit{low}}}}\,-u_{_{{\textit{high}}}}) + u_{_{{\textit{high}}}}$, and let *F* and *V* be the following sub-matrices of the Jacobian of the above system, evaluated at the solution ${\mathbf {w}}_{\small {{dfe}}}$

$$\begin{aligned} F= \begin{pmatrix} \frac{ \partial {\mathcal {F}}_i}{\partial x_j} \Big |_{{\mathbf {w}}_{\small {{dfe}}}} \\ \end{pmatrix}_{1 \le i,j \le 5} = \begin{pmatrix} 0 &{}\quad 0 &{}\quad 0 &{}\quad 0 &{}\quad \sigma \beta _{{M}} \\ 0 &{}\quad 0 &{}\quad 0 &{}\quad 0 &{}\quad 0 \\ 0 &{}\quad 0 &{}\quad 0 &{}\quad 0 &{}\quad 0 \\ 0 &{}\quad \sigma \beta _Y \frac{\varOmega _{{M}}}{\varOmega _{{H}}} \frac{\xi _{{H}}}{\xi _{{M}}}&{}\quad \sigma \beta _A \frac{\varOmega _{{M}}}{\varOmega _{{H}}} \frac{\xi _{{H}}}{\xi _{{M}}} &{}\quad 0 &{}\quad 0 \\ 0 &{}\quad 0 &{}\quad 0 &{}\quad 0 &{}\quad 0 \\ \end{pmatrix} \end{aligned}$$

and

$$\begin{aligned} V= \begin{pmatrix} \frac{ \partial {\mathcal {V}}_i}{\partial x_j} \Big |_{{\mathbf {w}}_{\small {{dfe}}}} \\ \end{pmatrix}_{1 \le i,j \le 5} = \begin{pmatrix} (\gamma +\xi _{{H}}) &{}\quad 0 &{}\quad 0 &{}\quad 0 &{}\quad 0\\ \gamma \left( U_{_{{\textit{low}}}} -1 \right) &{}\quad \left( \xi _{{H}} +\delta + \lambda _{YR}\right) &{}\quad 0 &{}\quad 0 &{}\quad 0 \\ -\gamma U_{_{{\textit{low}}}} &{}\quad 0 &{}\quad \left( \lambda _{AR}+\xi _{{H}}\right) &{}\quad 0 &{}\quad 0 \\ 0 &{}\quad 0 &{}\quad 0 &{}\quad (\xi _{{M}}+\tau ) &{}\quad 0 \\ 0 &{}\quad 0 &{}\quad 0 &{}\quad -\tau &{}\quad \xi _{{M}} \\ \end{pmatrix}. \end{aligned}$$

$$\begin{aligned} V^{-1 }= \begin{pmatrix} (\gamma +\xi _{{H}})^{-1} &{}\quad 0 &{}\quad 0 &{}\quad 0 &{}\quad 0\\ -\frac{\gamma \left( U_{{\textit{low}}}-1 \right) }{(\gamma +\xi _{{H}})\left( \xi _{{H}} +\delta + \lambda _{YR}\right) } &{}\quad \left( \xi _{{H}} +\delta + \lambda _{YR}\right) ^{-1} &{}\quad 0 &{}\quad 0 &{}\quad 0 \\ \frac{\gamma U_{{\textit{low}}}}{(\gamma +\xi _{{H}}) \left( \lambda _{AR}+\xi _{{H}}\right) } &{}\quad 0 &{}\quad \left( \lambda _{AR}+\xi _{{H}}\right) ^{-1} &{}\quad 0 &{}\quad 0 \\ 0 &{}\quad 0 &{}\quad 0 &{}\quad (\xi _{{M}}+\tau )^{-1} &{}\quad 0 \\ 0 &{}\quad 0 &{}\quad 0 &{}\quad \frac{\tau }{\xi _{{M}}(\xi _{{M}}+\tau )}&{}\quad \xi _{{M}}^{-1} \\ \end{pmatrix} \end{aligned}$$

and $FV^{-1}$ is given by the following matrix:

$$\begin{aligned} \begin{pmatrix} 0 &{}\quad 0 &{}\quad 0 &{}\quad \frac{\sigma \beta _{{M}} \tau }{\xi _{{M}}(\xi _{{M}}+\tau )}&{}\quad \frac{\sigma \beta _{{M}}}{\xi _{{M}}}\\ 0 &{}\quad 0 &{}\quad 0 &{}\quad 0 &{}\quad 0\\ 0 &{}\quad 0 &{}\quad 0 &{}\quad 0 &{}\quad 0\\ \frac{\sigma \gamma \varOmega _{{M}} \xi _{{H}}}{(\gamma + \xi _{{H}})\varOmega _{{H}} \xi _{{M}}}\left( \frac{\beta _A U_{_{{\textit{low}}}}}{\lambda _{AR}+\xi _{{H}}} - \frac{\beta _Y\left( U_{_{{\textit{low}}}} - 1\right) }{\xi _{{H}} +\delta + \lambda _{YR}}\right) &{}\quad \frac{\sigma \beta _Y \varOmega _{{M}} \xi _{{H}}}{(\xi _{{H}} + \delta + \lambda _{YR})\varOmega _{{H}} \xi _{{M}}} &{}\quad \frac{\sigma \beta _A \varOmega _{{M}} \xi _{{H}}}{(\xi _{{H}} + \lambda _{AR})\varOmega _{{H}} \xi _{{M}}} &{}\quad 0 &{}\quad 0 \\ 0 &{}\quad 0 &{}\quad 0 &{}\quad 0 &{}\quad 0\\ \end{pmatrix}. \end{aligned}$$

Let ${\mathcal {I}}$ denote the $5 \times 5$ identity matrix, so that the characteristic polynomial $P(\lambda )$ of the matrix $FV^{-1}$ is given by

$$\begin{aligned} P(\lambda )&= \det {\left( FV^{-1}-\lambda {\mathcal {I}} \right) },\\&=\lambda ^3 \left( \lambda ^2 - \left( \frac{\sigma ^2 \tau \gamma \varOmega _{{M}} \xi _{{H}} \beta _{{M}}}{\xi _{{M}}^2(\gamma + \xi _{{H}})(\tau + \xi _{{M}}) \varOmega _{{H}}} \left( \frac{\beta _AU_{_{{\textit{low}}}} }{\lambda _{AR} + \xi _{{H}}} - \frac{\beta _Y(U_{_{{\textit{low}}}}\,-1)}{\lambda _{YR} + \xi _{{H}} + \delta } \right) \right) \right) . \end{aligned}$$

The solution set $\lbrace \lambda _i \rbrace _{1 \le i \le 5}$ is given by

$$\begin{aligned} \left\{ 0,0,0, \pm \sqrt{\frac{\sigma ^2 \tau \gamma \varOmega _{{M}} \xi _{{H}} \beta _{{M}}}{\xi _{{M}}^2(\gamma + \xi _{{H}})(\tau + \xi _{{M}})\varOmega _{{H}}} \left( \frac{\beta _AU_{_{{\textit{low}}}} }{\lambda _{AR} + \xi _{{H}}} - \frac{\beta _Y(U_{_{{\textit{low}}}}\,-1)}{\lambda _{YR} + \xi _{{H}} + \delta } \right) }\right\} . \end{aligned}$$

Therefore, the reproductive threshold for the $SEY AR$ model (4) is given by

$$\begin{aligned} {\mathcal {R}}_0&:=\rho \left( FV^{-1}\right) , \\&=\max \limits _{1 \le i \le 5} {\lbrace \lambda _i \rbrace }, \\&=\sqrt{\frac{\sigma ^2 \tau \gamma \varOmega _{{M}} \xi _{{H}} \beta _{{M}}}{\xi _{{M}}^2(\gamma + \xi _{{H}})(\tau + \xi _{{M}})\varOmega _{{H}}} \left( \frac{\beta _AU_{_{{\textit{low}}}} }{\lambda _{AR} + \xi _{{H}}} - \frac{\beta _Y(U_{_{{\textit{low}}}}\,-1)}{\lambda _{YR} + \xi _{{H}} + \delta } \right) }.\\ \end{aligned}$$

The proof of the lemma regarding the local asymptotic stability (LAS) of the DFE ${\mathbf {w}}_{\small {{dfe}}}$ corresponding to the $SEY AR$ model (4) is now complete after invoking Theorem (6) in “Summary of Stability Theorems” of appendix. $\square$

#### Proof of Corollary 1 (See Page 18)

##### Proof

Let the DFE ${\mathbf {v}}_{\small {{dfe}}}$ be defined as

$$\begin{aligned} {\mathbf {v}}_{\small {{dfe}}}=\left( 0,0,0,0,0,\frac{\varOmega _{{H}}}{\xi _{{H}}},\frac{\varOmega _{{M}}}{\xi _{{M}}}\right) ^T. \end{aligned}$$

A straightforward calculation shows that the sub-matrices *F* and *V* of the Jacobian evaluated at the DFE ${\mathbf {v}}_{\small {{dfe}}}$ corresponding to Fig. 2 are given by

$$\begin{aligned} F= \begin{pmatrix} \frac{ \partial {\mathcal {F}}_i}{\partial x_j} \Big |_{{\mathbf {v}}_{\small {{dfe}}}} \\ \end{pmatrix}_{1 \le i,j \le 5} = \begin{pmatrix} 0 &{}\quad 0 &{}\quad 0 &{}\quad 0 &{}\quad \sigma \beta _{{M}} \\ 0 &{}\quad 0 &{}\quad 0 &{}\quad 0 &{}\quad 0 \\ 0 &{}\quad 0 &{}\quad 0 &{}\quad 0 &{}\quad 0 \\ 0 &{}\quad \sigma \beta _Y \frac{\varOmega _{{M}}}{\varOmega _{{H}}} \frac{\xi _{{H}}}{\xi _{{M}}}&{}\quad \sigma \beta _A \frac{\varOmega _{{M}}}{\varOmega _{{H}}} \frac{\xi _{{H}}}{\xi _{{M}}} &{}\quad 0 &{}\quad 0 \\ 0 &{}\quad 0 &{}\quad 0 &{}\quad 0 &{}\quad 0 \\ \end{pmatrix} \end{aligned}$$

and

$$\begin{aligned} V= \begin{pmatrix} \frac{ \partial {\mathcal {V}}_i}{\partial x_j} \Big |_{{\mathbf {v}}_{\small {{dfe}}}} \\ \end{pmatrix}_{1 \le i,j \le 5} = \begin{pmatrix} (\gamma +\xi _{{H}}) &{}\quad 0 &{}\quad 0 &{}\quad 0 &{}\quad 0\\ \gamma \left( U_{_{{\textit{low}}}} -1 \right) &{}\quad \left( \xi _{{H}} +\delta + \lambda _{YS}\right) &{}\quad 0 &{}\quad 0 &{}\quad 0 \\ -\gamma U_{_{{\textit{low}}}} &{}\quad 0 &{}\quad \left( \lambda _{AS}+\xi _{{H}}\right) &{}\quad 0 &{}\quad 0 \\ 0 &{}\quad 0 &{}\quad 0 &{}\quad (\xi _{{M}}+\tau ) &{}\quad 0 \\ 0 &{}\quad 0 &{}\quad 0 &{}\quad -\tau &{}\quad \xi _{{M}} \\ \end{pmatrix}. \end{aligned}$$

Therefore, the proof directly follows as in Corollary 2. $\square$

#### Proof of Corollary 2 (See Page 20)

##### Proof

A straightforward calculation shows that the sub-matrices *F* and *V* of the Jacobian evaluated at the DFE ${\mathbf {w}}_{\small {{dfe}}}$ corresponding to Fig. 3 are given by

$$\begin{aligned} F= \begin{pmatrix} \frac{ \partial {\mathcal {F}}_i}{\partial x_j} \Big |_{{\mathbf {w}}_{\small {{dfe}}}} \\ \end{pmatrix}_{1 \le i,j \le 5} = \begin{pmatrix} 0 &{}\quad 0 &{}\quad 0 &{}\quad 0 &{}\quad \sigma \beta _{{M}} \\ 0 &{}\quad 0 &{}\quad 0 &{}\quad 0 &{}\quad 0 \\ 0 &{}\quad 0 &{}\quad 0 &{}\quad 0 &{}\quad 0 \\ 0 &{}\quad \sigma \beta _Y \frac{\varOmega _{{M}}}{\varOmega _{{H}}} \frac{\xi _{{H}}}{\xi _{{M}}}&{}\quad \sigma \beta _A \frac{\varOmega _{{M}}}{\varOmega _{{H}}} \frac{\xi _{{H}}}{\xi _{{M}}} &{}\quad 0 &{}\quad 0 \\ 0 &{}\quad 0 &{}\quad 0 &{}\quad 0 &{}\quad 0 \\ \end{pmatrix} \end{aligned}$$

and

$$\begin{aligned} V= \begin{pmatrix} \frac{ \partial {\mathcal {V}}_i}{\partial x_j} \Big |_{{\mathbf {w}}_{\small {{dfe}}}} \\ \end{pmatrix}_{1 \le i,j \le 5} = \begin{pmatrix} (\gamma +\xi _{{H}}) &{}\quad 0 &{}\quad 0 &{}\quad 0 &{}\quad 0\\ \gamma \left( U_{_{{\textit{low}}}} -1 \right) &{}\quad \left( \xi _{{H}} +\delta + \lambda _{YR}\right) &{}\quad 0 &{}\quad 0 &{}\quad 0 \\ -\gamma U_{_{{\textit{low}}}} &{}\quad 0 &{}\quad \left( \lambda _{AR}+\xi _{{H}}\right) &{}\quad 0 &{}\quad 0 \\ 0 &{}\quad 0 &{}\quad 0 &{}\quad (\xi _{{M}}+\tau ) &{}\quad 0 \\ 0 &{}\quad 0 &{}\quad 0 &{}\quad -\tau &{}\quad \xi _{{M}} \\ \end{pmatrix}. \end{aligned}$$

The reproductive threshold ${\mathcal {R}}_0 :=\rho \left( FV^{-1}\right)$ for any given compartmentalized infectious disease model is completely determined by the matrices *F* and *V*. Therefore, it is of trivial consequence that the models corresponding to Figs. 1 and 3 possess identical reproductive thresholds, given by (7) arising from Lemma 1. $\square$

#### Proof of Theorem 4 (See Page 21)

##### Proof

Let $\varTheta _0$, ${\tilde{\varTheta }}_0$, ${\mathcal {R}}_A$, and ${\mathcal {R}}_Y$ be defined as in the statement of the theorem. Additionally, denote the following quantities $r^0_6:=r_6\Big |_{\varTheta _0}$, $C^0_1:=C_1\Big |_{\varTheta _0}$, $C^0_2:=C_2\Big |_{{\tilde{\varTheta }}_0}$ and $C^0_0:=C_0\Big |_{\varTheta _0}=C_0\Big |_{{\tilde{\varTheta }}_0}$, where the terms $r_6$ and $C_i$ for $i=0,1,2$ are defined as in Sect. 3.2. By the monotonicity of the square foot function, it follows that

$$\begin{aligned} {\mathcal {R}}_A&:={\mathcal {R}}_0\Big |_{\varTheta _0}= C^0_0 \sqrt{r^0_6}> C^0_0\sqrt{r^0_6-U_{{_{{\textit{low}}}}_{_0}}C^0_1}= C^0_0\sqrt{\left( 1-U_{{_{{\textit{low}}}}_{_0}}\right) C^0_2}={\mathcal {R}}_0\Big |_{{\tilde{\varTheta }}_0}:={\mathcal {R}}_Y. \end{aligned}$$

$$\square $$

#### Proof of Theorem 5 (See Page 25)

##### Proof

If we consider the dynamical system ${\dot{x}}=g(x,\omega )$, where $\omega$ is a bifurcation parameter and the vector field *g* is $C^2$ in both *x* and $\omega$, then the disease-free equilibrium can be viewed as the manifold $({\mathbf {x}}_{\small {{dfe}}};\omega )$ where the local stability of ${\mathbf {x}}_{\small {{dfe}}}$ changes at the point $({\mathbf {x}}_{\small {{dfe}}};\omega ^\star )$. Now we shall investigate the existence and stability of non-trivial equilibrium states in a neighborhood of the bifurcation point. We focus on the disease-free equilibrium ${\mathbf {x}}_{\small {{dfe}}}$ and study the occurrence of a transcritical bifurcation at ${\mathcal {R}}_0=1$. Since ${\mathcal {R}}_0$ consists of the square root of a complicated combination of parameters, it is not practical to use as a bifurcation parameter. However, observe that ${\mathcal {R}}_0=1$ if and only if

$$\begin{aligned} \beta _{{M}}&=\frac{\xi _{{M}}^2 \varOmega _{{H}} (\gamma + \xi _{{H}})(\tau + \xi _{{M}})(\lambda _{AR} + \xi _{{H}})(\lambda _{YR} + \xi _{{H}} + \delta )}{\sigma ^2 \tau \xi _{{H}} \varOmega _{{M}} \left( \gamma U_{_{{\textit{low}}}} \beta _A (\lambda _{YR} + \xi _{{H}} + \delta )- \gamma (U_{_{{\textit{low}}}}\,-1) \beta _Y(\lambda _{AR} + \xi _{{H}}) \right) },\\&:= \beta _{{M}}^\star . \end{aligned}$$

In lieu of Lemma 1, it follows that ${\mathbf {x}}_{\small {{dfe}}}$ is locally asymptotically stable when $\beta _{{M}}<\beta _{{M}}^\star$ and unstable if $\beta _{{M}}>\beta _{{M}}^\star$. Thus, the combination of parameters $\beta _{{M}}^\star$ is a bifurcation value. To simplify the notation, we rewrite system (4) as ${\dot{x}}=g(x,\beta _{{M}})$ where ${\mathbf {x}}=\left( S,E,Y,A,R,M_{{S}},M_{{E}},M_{{I}}\right) ^T$ so that $x_i$ is the $i\text {th}$ component of ${\mathbf {x}}$ and $g=(g_1,g_2,g_3,g_4,g_5,g_6,g_7,g_8)^T$, or more explicitly

14 $$\begin{aligned} g_1&=\varOmega _{{H}} + \lambda _{RS} x_5 - \left( \sigma \beta _{{M}} \frac{x_8}{\sum _i^5 x_i} + \xi _{{H}} \right) x_1,\nonumber \\ g_2&=\sigma \beta _{{M}} \frac{x_8}{\sum _i^5 x_i}x_1- (\gamma +\xi _{{H}})x_2,\nonumber \\ g_3&=\gamma \left( 1-u(\varepsilon )\right) x_2 - \left( \xi _{{H}} +\delta + \lambda _{YR}\right) x_3,\nonumber \\ g_4&=\gamma u(\varepsilon ) x_2 - \left( \lambda _{AR}+\xi _{{H}}\right) x_4, \\ g_5&=\lambda _{AR}x_4 + \lambda _{YR}x_3 - \left( \lambda _{RS} +\xi _{{H}}\right) x_5,\nonumber \\ g_6&=\varOmega _{{M}} - \left( \xi _{{M}} + \sigma \beta _Y \frac{x_3}{\sum _i^5 x_i} +\sigma \beta _A \frac{x_4}{\sum _i^5 x_i} \right) x_6, \nonumber \\ g_7&=\sigma \left( \beta _Y \frac{x_3}{\sum _i^5 x_i} +\beta _A \frac{x_4}{\sum _i^5 x_i}\right) x_6 - \left( \xi _{{M}} + \tau \right) x_7, \nonumber \\ g_8&=\tau x_7- \xi _{{M}} x_8.\nonumber \end{aligned}$$

Denote $J({\mathbf {x}}_{\small {{dfe}}},\beta _{{M}}^\star )$ to be the Jacobian of *g* evaluated at the DFE ${\mathbf {x}}_{\small {{dfe}}}$ and threshold $\beta _{{M}}^\star$. Thus, $J({\mathbf {x}}_{\small {{dfe}}},\beta _{{M}}^\star )$ is given by

$$\begin{aligned} \begin{pmatrix} -\xi _{{H}} &{}\quad 0 &{}\quad 0 &{}\quad 0 &{}\quad \epsilon &{}\quad 0 &{}\quad 0 &{}\quad -\sigma \beta _{{M}}^\star \\ 0 &{}\quad -(\gamma + \xi _{{H}}) &{}\quad 0 &{}\quad 0 &{}\quad 0 &{}\quad 0 &{}\quad 0 &{}\quad \sigma \beta _{{M}}^\star \\ 0 &{}\quad \gamma (1-U_{_{{\textit{low}}}}) &{}\quad -(\delta + \lambda _{YR} + \xi _{{H}}) &{}\quad 0 &{}\quad 0 &{}\quad 0 &{}\quad 0 &{}\quad 0\\ 0 &{}\quad \gamma U_{_{{\textit{low}}}} &{}\quad 0 &{}\quad -(\lambda _{AR} + \xi _{{H}})&{}\quad 0 &{}\quad 0 &{}\quad 0 &{}\quad 0\\ 0 &{}\quad 0 &{}\quad \lambda _{YR} &{}\quad \lambda _{AR} &{}\quad -(\lambda _{RS} + \xi _{{H}}) &{}\quad 0 &{}\quad 0 &{}\quad 0\\ 0 &{}\quad 0 &{}\quad -\frac{\sigma \beta _Y \xi _{{H}} \varOmega _{{M}}}{\varOmega _{{H}} \xi _{{M}}} &{}\quad - \frac{\sigma \beta _A \xi _{{H}} \varOmega _{{M}}}{\varOmega _{{H}} \xi _{{M}}} &{}\quad 0 &{}\quad - \xi _{{M}} &{}\quad 0 &{}\quad 0\\ 0 &{}\quad 0 &{}\quad \frac{\sigma \beta _Y \xi _{{H}} \varOmega _{{M}}}{\varOmega _{{H}} \xi _{{M}}} &{}\quad \frac{\sigma \beta _A \xi _{{H}} \varOmega _{{M}}}{\varOmega _{{H}} \xi _{{M}}} &{}\quad 0 &{}\quad 0 &{}\quad -(\tau + \xi _{{M}}) &{}\quad 0\\ 0 &{}\quad 0 &{}\quad 0 &{}\quad 0 &{}\quad 0 &{}\quad 0 &{}\quad \tau &{}\quad -\xi _{{M}}\\ \end{pmatrix}. \end{aligned}$$

If we invoke the following positive change of variables

$$\begin{aligned} {\left\{ \begin{array}{ll} K_1 &{}= \gamma + \xi _{{H}},\\ K_2 &{}= \gamma (1-U_{_{{\textit{low}}}}),\\ K_3 &{}= \gamma U_{_{{\textit{low}}}},\\ K_4 &{}= \delta + \lambda _{YR} + \xi _{{H}},\\ K_5 &{}= \lambda _{AR} + \xi _{{H}},\\ K_6 &{}= \lambda _{RS} + \xi _{{H}},\\ K_7 &{}= \tau + \xi _{{M}},\\ \end{array}\right. } \end{aligned}$$

then $\beta _{{H}}^\star$ can be written as

$$\begin{aligned} \beta _{{M}}^\star =\frac{\xi _{{M}}^2 \varOmega _{{H}} K_1 K_4 K_5 K_7}{\sigma ^2 \tau \xi _{{H}} \varOmega _{{M}} \left( \beta _A K_3 K_4 + \beta _Y K_2 K_5 \right) }. \end{aligned}$$

As a result, $J({\mathbf {x}}_{\small {{dfe}}},\beta _{{M}}^\star )$ can be written as

$$\begin{aligned} \begin{pmatrix} -\xi _{{H}} &{}\quad 0 &{}\quad 0 &{}\quad 0 &{}\quad \epsilon &{}\quad 0 &{}\quad 0 &{}\quad -\frac{\xi _{{M}}^2 \varOmega _{{H}} K_1 K_4 K_5 K_7}{\sigma \tau \xi _{{H}} \varOmega _{{M}} \left( \beta _A K_3 K_4 + \beta _Y K_2 K_5 \right) }\\ 0 &{}\quad -K_1 &{}\quad 0 &{}\quad 0 &{}\quad 0 &{}\quad 0 &{}\quad 0 &{}\quad \frac{\xi _{{M}}^2 \varOmega _{{H}} K_1 K_4 K_5 K_7}{\sigma \tau \xi _{{H}} \varOmega _{{M}} \left( \beta _A K_3 K_4 + \beta _Y K_2 K_5 \right) }\\ 0 &{}\quad K_2 &{}\quad -K_4 &{}\quad 0 &{}\quad 0 &{}\quad 0 &{}\quad 0 &{}\quad 0\\ 0 &{}\quad K_3 &{}\quad 0 &{}\quad -K_5 &{}\quad 0 &{}\quad 0 &{}\quad 0 &{}\quad 0\\ 0 &{}\quad 0 &{}\quad \lambda _{YR} &{}\quad \lambda _{AR} &{}\quad -K_6 &{}\quad 0 &{}\quad 0 &{}\quad 0\\ 0 &{}\quad 0 &{}\quad -\frac{\sigma \beta _Y \xi _{{H}} \varOmega _{{M}}}{\varOmega _{{H}} \xi _{{M}}} &{}\quad -\frac{\sigma \beta _A \xi _{{H}} \varOmega _{{M}}}{\varOmega _{{H}} \xi _{{M}}} &{}\quad 0 &{}\quad - \xi _{{M}} &{}\quad 0 &{}\quad 0\\ 0 &{}\quad 0 &{}\quad \frac{\sigma \beta _Y \xi _{{H}} \varOmega _{{M}}}{\varOmega _{{H}} \xi _{{M}}} &{}\quad \frac{\sigma \beta _A \xi _{{H}} \varOmega _{{M}}}{\varOmega _{{H}} \xi _{{M}}} &{}\quad 0 &{}\quad 0 &{}\quad -K_7 &{}\quad 0\\ 0 &{}\quad 0 &{}\quad 0 &{}\quad 0 &{}\quad 0 &{}\quad 0 &{}\quad \tau &{}\quad -\xi _{{M}}\\ \end{pmatrix}. \end{aligned}$$

Due to the equivalency of the two conditions ${\mathcal {R}}_0=1$ and $\beta _{{M}}=\beta _{{M}}^\star$, it follows that $J({\mathbf {x}}_{\small {{dfe}}},\beta _{{M}}^\star )$ contains information of the linearized system evaluated at the disease-free equilibrium and threshold value. Utilizing the machinery covered in “Summary of Stability Theorems” of appendix below, the Jacobian evaluated at the threshold value, i.e., $J({\mathbf {x}}_{\small {{dfe}}},\beta _{{M}}^\star )$, has a zero simple eigenvalue with all others having negative real parts. Therefore, the hypothesis of Theorem 7 is satisfied. We proceed by calculating the *a* and *b* terms (18) and (19) appearing in Theorem 7. In observance of the conclusions in the theorem, it follows that the $SEY AR$ model (4) will undergo a super-critical bifurcation if $a>0$ and $b>0$ and a sub-critical bifurcation if $a<0$ and $b>0$. The main ingredients in calculating *a* and *b* are the generalized right and left eigenvectors of the matrix $J({\mathbf {x}}_{\small {{dfe}}},\beta _{{M}}^\star )$ and their corresponding nonzero Hessian entries, evaluated at the DFE ${\mathbf {x}}_{\small {{dfe}}}$. In this fashion, we let $w =\left( w_1,w_2,w_3,w_4,w_5,w_6,w_7,w_8\right)$ and $v^T=\left( v_1,v_2,v_3,v_4,v_5,v_6,v_7,v_8\right) ^T$ be right and left generalized eigenvectors of $J({\mathbf {x}}_{\small {{dfe}}},\beta _{{M}}^\star )$, respectively. Since *J* is not symmetric, the left and right generalized eigenspaces are not equivalent. Solving the equations $Jw=0$ and $\left( v^TJ\right) ^T=J^Tv=0$, where $D_i \in {\mathbb {R}}_{\tiny {>0}}$ for $i=1,2$, yields

$$\begin{aligned} \begin{pmatrix} w_1\\ w_2\\ w_3\\ w_4\\ w_5\\ w_6\\ w_7\\ w_8\\ \end{pmatrix} = D_1\begin{pmatrix} \frac{\epsilon \lambda _{AR} K_3 K_4 +\epsilon \lambda _{YR} K_2 K_5 - K_1 K_6 K_4 K_5}{K^2_6 K_4 K_5}\\ 1\\ \frac{K_2}{K_4}\\ \frac{K_3}{K_5}\\ \frac{\lambda _{YR} K_2 K_5 + \lambda _{AR} K_3 K_4}{K_4 K_5 K_6}\\ -\frac{\sigma \xi _{{H}} \varOmega _{{M}}(\beta _Y K_2 K_5 + \beta _A K_3 K_4)}{\varOmega _{{H}} \xi ^2_{{M}} K_4 K_5}\\ \frac{\sigma \xi _{{H}} \varOmega _{{M}} \left( \beta _A K_3 K_4 + \beta _Y K_2 K_5\right) }{\xi _{{M}} \varOmega _{{H}} K_4 K_5 K_7}\\ \frac{\sigma \tau \xi _{{H}} \varOmega _{{M}} \left( \beta _A K_3 K_4 + \beta _Y K_2 K_5\right) }{\xi ^2_{{M}} \varOmega _{{H}} K_4 K_5 K_7}\\ \end{pmatrix} \end{aligned}$$

$$\begin{aligned} \begin{pmatrix} v_1\\ v_2\\ v_3\\ v_4\\ v_5\\ v_6\\ v_7\\ v_8\\ \end{pmatrix} = D_2\begin{pmatrix} 0\\ \frac{\sigma \tau \xi _{{H}} \varOmega _{{M}} \left( \beta _A K_3 K_4 + \beta _Y K_2 K_5\right) }{\xi _{{M}} \varOmega _{{H}} K_1 K_4 K_5 K_7}\\ \frac{\sigma \tau \beta _Y \xi _{{H}} \varOmega _{{M}}}{\varOmega _{{H}} \xi _{{M}} K_4 K_7}\\ \frac{\sigma \tau \beta _A \xi _{{H}} \varOmega _{{M}}}{\varOmega _{{H}} \xi _{{M}} K_5 K_7}\\ 0\\ 0\\ \frac{\tau }{K_7}\\ 1\\ \end{pmatrix}. \end{aligned}$$

For system (14), the nonzero partial derivatives of *g* evaluated at ${\mathbf {x}}_{\small {{dfe}}}$ are

$$\begin{aligned} \frac{\partial ^2 g_1}{\partial x_8 \partial x_2}&= \frac{\partial ^2 g_1}{\partial x_8 \partial x_3}=\frac{\partial ^2 g_1}{\partial x_8 \partial x_4}=\frac{\partial ^2 g_1}{\partial x_8 \partial x_5}=\frac{\sigma \beta _{{M}} \xi _{{H}}}{\varOmega _{{H}}},\\ \frac{\partial ^2 g_2}{\partial x_8 \partial x_2}&= \frac{\partial ^2 g_2}{\partial x_8 \partial x_3}=\frac{\partial ^2 g_2}{\partial x_8 \partial x_4}=\frac{\partial ^2 g_2}{\partial x_8 \partial x_5}=-\frac{\sigma \beta _{{M}} \xi _{{H}}}{\varOmega _{{H}}},\\ \frac{\partial ^2 g_3}{\partial x_2 \partial x_2}&= \frac{2 \gamma \xi _{{H}} e^{\varepsilon _0}}{\varOmega _{{H}}}(u_{_{{\textit{low}}}}\,-u_{_{{\textit{high}}}}), \quad \frac{\partial ^2 g_4}{\partial x_2 \partial x_2} = \frac{2 \gamma \xi _{{H}} e^{\varepsilon _0}}{\varOmega _{{H}}} (u_{_{{\textit{high}}}}-u_{_{{\textit{low}}}}),\\ \frac{\partial ^2 g_6}{\partial x_3 \partial x_1}&= \frac{\partial ^2 g_6}{\partial x_3 \partial x_2}=\frac{\partial ^2 g_6}{\partial x_3 \partial x_5}=\frac{\sigma \beta _Y \xi ^2_{{H}} \varOmega _{{M}}}{\varOmega ^2_{{H}} \xi _{{M}}},\\ \frac{\partial ^2 g_6}{\partial x_4 \partial x_1}&= \frac{\partial ^2 g_6}{\partial x_4 \partial x_2}=\frac{\partial ^2 g_6}{\partial x_4 \partial x_5}=\frac{\sigma \beta _A \xi ^2_{{H}} \varOmega _{{M}}}{\varOmega ^2_{{H}} \xi _{{M}}},\\ \frac{\partial ^2 g_6}{\partial x_3 \partial x_3}&= 2\frac{\partial ^2 g_6}{\partial x_3 \partial x_1},\quad \frac{\partial ^2 g_6}{\partial x_4 \partial x_4} = 2\frac{\partial ^2 g_6}{\partial x_4 \partial x_1},\\ \frac{\partial ^2 g_6}{\partial x_6 \partial x_3}&= -\frac{\sigma \beta _Y \xi _{{H}}}{\varOmega _{{H}}}, \frac{\partial ^2 g_6}{\partial x_6 \partial x_4} = -\frac{\sigma \beta _A \xi _{{H}}}{\varOmega _{{H}}},\\ \frac{\partial ^2 g_6}{\partial x_3 \partial x_4}&= \frac{\sigma \xi ^2_{{H}} \varOmega _{{M}} (\beta _Y +\beta _A)}{\xi _{{M}} \varOmega ^2_{{H}}},\\ \frac{\partial ^2 g_7}{\partial x_3 \partial x_1}&= \frac{\partial ^2 g_7}{\partial x_3 \partial x_2}=\frac{\partial ^2 g_7}{\partial x_3 \partial x_5}=-\frac{\sigma \beta _Y \xi ^2_{{H}} \varOmega _{{M}}}{\varOmega ^2_{{H}} \xi _{{M}}},\\ \frac{\partial ^2 g_7}{\partial x_4 \partial x_1}&= \frac{\partial ^2 g_7}{\partial x_4 \partial x_2}=\frac{\partial ^2 g_7}{\partial x_4 \partial x_5}=-\frac{\sigma \beta _A \xi ^2_{{H}} \varOmega _{{M}}}{\varOmega ^2_{{H}} \xi _{{M}}},\\ \frac{\partial ^2 g_7}{\partial x_3 \partial x_3}&= 2\frac{\partial ^2 g_7}{\partial x_3 \partial x_1},\quad \frac{\partial ^2 g_7}{\partial x_4 \partial x_4} = 2\frac{\partial ^2 g_7}{\partial x_4 \partial x_1},\\ \frac{\partial ^2 g_7}{\partial x_6 \partial x_3}&= \frac{\sigma \beta _Y \xi _{{H}}}{\varOmega _{{H}}},\quad \frac{\partial ^2 g_7}{\partial x_6 \partial x_4} = \frac{\sigma \beta _A \xi _{{H}}}{\varOmega _{{H}}},\\ \frac{\partial ^2 g_7}{\partial x_3 \partial x_4}&= -\frac{\sigma \xi ^2_{{H}} \varOmega _{{M}} (\beta _Y +\beta _A)}{\xi _{{M}} \varOmega ^2_{{H}}},\\ \frac{\partial ^2 g_1}{\partial x_8 \partial \beta _{{H}}}&= -\sigma ,\quad \frac{\partial ^2 g_2}{\partial x_8 \partial \beta _{{H}}} = \sigma . \end{aligned}$$

Due to the fact that the parameter $\beta _{{M}}$ only appears in system (14) twice, the calculation of the *b* term is less involved. Indeed, for *b* we have

15 $$\begin{aligned} b&=\sum _{i,k=1}^{8} v_k w_i \frac{\partial ^2 g_k}{\partial x_i \partial \beta _{{H}}}\left( {\mathbf {x}}_{\small {{dfe}}},\beta _{{M}}^\star \right) ,\nonumber \\&=v_1 w_8 \frac{\partial ^2 g_1}{\partial x_8 \partial \beta _{{M}}}\left( {\mathbf {x}}_{\small {{dfe}}},\beta _{{M}}^\star \right) + v_2 w_8 \frac{\partial ^2 g_2}{\partial x_8 \partial \beta _{{M}}}\left( {\mathbf {x}}_{\small {{dfe}}},\beta _{{M}}^\star \right) , \nonumber \\&= \sigma v_2 w_8, \nonumber \\&= \frac{\sigma ^2 \tau \xi _{{H}} \varOmega _{{M}} \left( \beta _A K_3 K_4 + \beta _Y K_2 K_5\right) }{\xi _{{M}} \varOmega _{{H}} K_1 K_4 K_5 K_7}v_8 w_8, \nonumber \\&= \frac{\sigma ^3 \tau ^2 \xi ^2_{{H}} \varOmega ^2_{{M}} \left( \beta _A K_3 K_4 + \beta _Y K_2 K_5\right) ^2}{\xi ^3_{{M}} \varOmega ^2_{{H}} K_1 K^2_4 K^2_5 K^2_7} w_2 v_8, \nonumber \\&= \frac{\sigma ^3 \tau ^2 \xi ^2_{{H}} \varOmega ^2_{{M}} \left( \beta _A K_3 K_4 + \beta _Y K_2 K_5\right) ^2}{\xi ^3_{{M}} \varOmega ^2_{{H}} K_1 K^2_4 K^2_5 K^2_7} D_1 D_2, \nonumber \\&>0. \end{aligned}$$

Since $D_i \in {\mathbb {R}}_{\tiny {>0}}$ for $i=1,2$, it follows that *b* is always positive; hence, the bifurcation behavior of system (14) is completely determined by the sign of *a*. To simplify the pending calculation, we invoke the following change of variables:

$$\begin{aligned} {\left\{ \begin{array}{ll} Z_1 &{}= \frac{\sigma \beta _{{M}} \xi _{{H}}}{\varOmega _{{H}}},\\ Z_2 &{}= \frac{\sigma \beta _Y \xi ^2_{{H}} \varOmega _{{M}}}{\varOmega ^2_{{H}} \xi _{{M}}},\\ Z_3 &{}= \frac{\sigma \beta _A \xi ^2_{{H}} \varOmega _{{M}}}{\varOmega ^2_{{H}} \xi _{{M}}},\\ Z_4 &{}= \frac{\sigma \xi ^2_{{H}} \varOmega _{{M}} (\beta _Y +\beta _A)}{\xi _{{M}} \varOmega ^2_{{H}}},\\ Z_5 &{}= \frac{\sigma \beta _A \xi _{{H}}}{\varOmega _{{H}}},\\ Z_6 &{}= \frac{2 \gamma \xi _{{H}} e^{\varepsilon _0}}{\varOmega _{{H}}} (u_{_{{\textit{high}}}}-u_{_{{\textit{low}}}}),\\ Z_7 &{}= \frac{\sigma \beta _Y \xi _{{H}}}{\varOmega _{{H}}},\\ Q_0 &{}= \frac{\lambda _{YR} K_2 K_5 + \lambda _{AR} K_3 K_4}{K_4 K_5 K_6},\\ Q_1 &{}= \frac{\beta _Y K_2 K_5 + \beta _A K_3 K_4}{K_4 K_5 K_7},\\ Q_2 &{}= \frac{\sigma \tau \xi _{{H}} \varOmega _{{M}} \beta _Y}{\varOmega _{{H}} \xi _{{M}} K_4 K_7},\\ Q_3 &{}= \frac{\sigma \tau \xi _{{H}} \varOmega _{{M}} \beta _A}{\varOmega _{{H}} \xi _{{M}} K_5 K_7},\\ Q_4 &{}= \frac{\sigma \xi _{{H}} \varOmega _{{M}}}{\varOmega _{{H}} \xi _{{M}}},\\ \end{array}\right. } \end{aligned}$$

so that the generalized eigenvectors can be written as:

$$\begin{aligned} \begin{pmatrix} w_1\\ w_2\\ w_3\\ w_4\\ w_5\\ w_6\\ w_7\\ w_8\\ \end{pmatrix} = D_1\begin{pmatrix} \frac{\lambda _{RS} Q_0-K_1}{K_6}\\ 1\\ \frac{K_2}{K_4}\\ \frac{K_3}{K_5}\\ Q_0\\ -\frac{Q_4 Q_1 K_7}{\xi _{{M}}}\\ Q_4 Q_1\\ \frac{\tau Q_4 Q_1}{\xi _{{M}}}\\ \end{pmatrix} \quad \mathrm{and} \quad \begin{pmatrix} v_1\\ v_2\\ v_3\\ v_4\\ v_5\\ v_6\\ v_7\\ v_8\\ \end{pmatrix} = D_2\begin{pmatrix} 0\\ \frac{\tau Q_4 Q_1}{K_1}\\ Q_2\\ Q_3\\ 0\\ 0\\ \frac{\tau }{K_7}\\ 1\\ \end{pmatrix}. \end{aligned}$$

In view of the relatively strong regularity assumptions mentioned in the beginning of Sect. 3.1 and natural symmetry of the system, it follows that $\frac{\partial ^2 g_k}{\partial x_i \partial x_j} v_k w_i w_j=\frac{\partial ^2 g_k}{\partial x_j \partial x_i} v_k w_j w_i$ for all $1 \le i,j \le 8$. Furthermore, $\frac{\partial ^2 g_k}{\partial x_i \partial x_j} v_k w_i w_j=0$ for $k=1,5,6,8$, since $\frac{\partial ^2 g_8}{\partial x_i \partial x_j}=0$ for all $1 \le i,j \le 8$ and $v_k=0$ for $k=1,5,6$. As a result, the terms that contribute to the sum correspond to $k=2,3,4,7$. Thus, *a* can be written as

16 $$\begin{aligned} a&= \sum _{i,j,k=1}^{8} v_k w_i w_j \frac{\partial ^2 g_k}{\partial x_i \partial x_j}\left( {\mathbf {x}}_{\small {{dfe}}},\beta _{{M}}^\star \right) \nonumber \\&= 2\Bigg [v_2 w_8 w_2 \frac{\partial ^2 g_2}{\partial x_8\partial x_2}\left( {\mathbf {x}}_{\small {{dfe}}},\beta _{{M}}^\star \right) + v_2 w_8 w_3 \frac{\partial ^2 g_2}{\partial x_8\partial x_3}\left( {\mathbf {x}}_{\small {{dfe}}},\beta _{{M}}^\star \right) \nonumber \\&\qquad \quad +\,v_2 w_8 w_4 \frac{\partial ^2 g_2}{\partial x_8\partial x_4}\left( {\mathbf {x}}_{\small {{dfe}}},\beta _{{M}}^\star \right) + v_2 w_8 w_5 \frac{\partial ^2 g_2}{\partial x_8\partial x_5}\left( {\mathbf {x}}_{\small {{dfe}}},\beta _{{M}}^\star \right) \nonumber \\&\qquad \quad +\,v_3 w^2_2 \frac{\partial ^2 g_3}{\partial x_2\partial x_2}\left( {\mathbf {x}}_{\small {{dfe}}},\beta _{{M}}^\star \right) +v_4 w^2_2 \frac{\partial ^2 g_4}{\partial x_2\partial x_2}\left( {\mathbf {x}}_{\small {{dfe}}},\beta _{{M}}^\star \right) \nonumber \\&\qquad \quad +\,v_7 w_3 w_1 \frac{\partial ^2 g_7}{\partial x_3\partial x_1}\left( {\mathbf {x}}_{\small {{dfe}}},\beta _{{M}}^\star \right) + v_7 w_3 w_2 \frac{\partial ^2 g_7}{\partial x_3\partial x_2}\left( {\mathbf {x}}_{\small {{dfe}}},\beta _{{M}}^\star \right) \nonumber \\&\qquad \quad +\, v_7 w_3 w_5 \frac{\partial ^2 g_7}{\partial x_3\partial x_5}\left( {\mathbf {x}}_{\small {{dfe}}},\beta _{{M}}^\star \right) + v_7 w_4 w_1 \frac{\partial ^2 g_7}{\partial x_4\partial x_1}\left( {\mathbf {x}}_{\small {{dfe}}},\beta _{{M}}^\star \right) \nonumber \\&\qquad \quad +\,v_7 w_4 w_2 \frac{\partial ^2 g_7}{\partial x_4\partial x_2}\left( {\mathbf {x}}_{\small {{dfe}}},\beta _{{M}}^\star \right) + v_7 w_4 w_5 \frac{\partial ^2 g_7}{\partial x_4\partial x_5}\left( {\mathbf {x}}_{\small {{dfe}}},\beta _{{M}}^\star \right) \nonumber \\&\qquad \quad +\,v_7 w^2_3\frac{\partial ^2 g_7}{\partial x_3\partial x_3}\left( {\mathbf {x}}_{\small {{dfe}}},\beta _{{M}}^\star \right) + v_7 w^2_4 \frac{\partial ^2 g_7}{\partial x_4\partial x_4}\left( {\mathbf {x}}_{\small {{dfe}}},\beta _{{M}}^\star \right) \nonumber \\&\qquad \quad +\,v_7 w_6 w_3 \frac{\partial ^2 g_7}{\partial x_6\partial x_3}\left( {\mathbf {x}}_{\small {{dfe}}},\beta _{{M}}^\star \right) +v_7 w_6 w_4 \frac{\partial ^2 g_7}{\partial x_6\partial x_4}\left( {\mathbf {x}}_{\small {{dfe}}},\beta _{{M}}^\star \right) \nonumber \\&\qquad \quad +\,v_7 w_3 w_4 \frac{\partial ^2 g_7}{\partial x_3\partial x_4}\left( {\mathbf {x}}_{\small {{dfe}}},\beta _{{M}}^\star \right) \Bigg ]\nonumber \\&= 2\Bigg [-Z_1 v_2 w_8 w_2 - Z_1 v_2 w_8 w_3 - Z_1 v_2 w_8 w_4 - Z_1 v_2 w_8 w_5 - Z_6 v_3 w^2_2 \nonumber \\&\qquad \quad +\,Z_6 v_4 w^2_2 - Z_2 v_7 w_3 w_1 -Z_2 v_7 w_3 w_2 - Z_2 v_7 w_3 w_5-Z_3 v_7 w_4 w_1 \nonumber \\&\qquad \quad -\,Z_3 v_7 w_4 w_2 - Z_3 v_7 w_4 w_5 - 2Z_2 v_7 w^2_3 - 2Z_3 v_7 w^2_4 +Z_7 v_7 w_6 w_3 \nonumber \\&\qquad \quad +\,Z_5 v_7 w_6 w_4 + Z_4 v_7 w_3 w_4 \Bigg ]\nonumber \\&= 2\Bigg [Z_7 v_7 w_6 w_3 + Z_5 v_7 w_6 w_4 + Z_4 v_7 w_3 w_4+Z_6 v_4 w^2_2-\Big (Z_1 v_2 w_8 w_2 \nonumber \\&\qquad \quad +\,Z_1 v_2 w_8 w_3 + Z_1 v_2 w_8 w_4 + Z_1 v_2 w_8 w_5 + Z_6 v_3 w^2_2 +Z_2 v_7 w_3 w_1 \nonumber \\&\qquad \quad +\,Z_2 v_7 w_3 w_2 + Z_2 v_7 w_3 w_5 + Z_3 v_7 w_4 w_1 + Z_3 v_7 w_4 w_2\nonumber \\&\qquad \quad +\,Z_3 v_7 w_4 w_5 + 2Z_2 v_7 w^2_3 + 2Z_3 v_7 w^2_4\Big )\Bigg ]. \end{aligned}$$

Upon grouping positive terms and simplifying, the right-hand side of the above expression can be written as:

$$\begin{aligned}&2 D_1^2 D_2 \Bigg [-\frac{\tau Z_7 Q_4 Q_1 K_2}{\xi _{{M}} K_4}-\frac{\tau Z_5 Q_4 Q_1 K_3}{\xi _{{M}} K_5}+\frac{\tau Z_4 K_2 K_3}{K_4 K_5 K_7} + Z_6 Q_3 \\&\qquad \qquad -\,\Bigg (Z_6 Q_2 + \frac{\tau ^2 Z_1 Q^2_1 Q^2_4}{K_1 \xi _{{M}}}\left( 1+ \frac{K_2}{K_4} + \frac{K_3}{K_5}+ Q_0\right) \\&\qquad \qquad +\frac{\tau Z_2 K_2}{K_4 K_7}\left( \frac{\lambda _{RS} Q_0-K_1}{K_6} + 1 + Q_0\right) \\&\qquad \qquad +\,\frac{\tau Z_3 K_3}{K_5 K_7}\left( \frac{\lambda _{RS}Q_0- K_1}{K_6} + 1 + Q_0\right) + \frac{2\tau Z_2 K^2_2}{K^2_4 K_7}+ \frac{2\tau Z_3 K^2_3}{K^2_5 K_7}\Bigg ) \Bigg ]\\&\quad =\, 2 D_1^2 D_2 \Bigg [\frac{\tau Z_2 K_1 K_2}{K_4 K_6 K_7}+\frac{\tau Z_3 K_1 K_3}{K_5 K_6 K_7}+\frac{\tau Z_4 K_2 K_3}{K_4 K_5 K_7} + Z_6 Q_3 -\Bigg (Z_6 Q_2 \nonumber \\&\qquad \qquad +\,\frac{\tau ^2 Z_1 Q^2_1 Q^2_4}{K_1 \xi _{{M}}}\left( 1+ \frac{K_2}{K_4} + \frac{K_3}{K_5}+ Q_0\right) +\frac{\tau Z_2 K_2}{K_4 K_7}\left( \frac{\lambda _{RS} Q_0}{K_6} + 1 + Q_0\right) \\&\qquad \qquad +\,\frac{\tau Z_3 K_3}{K_5 K_7}\left( \frac{\lambda _{RS} Q_0}{K_6} + 1 + Q_0\right) +\frac{\tau Z_7 Q_4 Q_1 K_2}{\xi _{{M}} K_4}+\frac{\tau Z_5 Q_4 Q_1 K_3}{\xi _{{M}} K_5} \\&\qquad \qquad + \frac{2\tau Z_2 K^2_2}{K^2_4 K_7}+ \frac{2\tau Z_3 K^2_3}{K^2_5 K_7}\Bigg ) \Bigg ]\\&\quad =\, 2 D_1^2 D_2 (\eta _2-\eta _1)\\&\quad =\, 2 D_1^2 D_2 (\frac{\eta _2}{\eta _1}-1)\eta _1, \end{aligned}$$

where

$$\begin{aligned} \eta _1&:=\, Z_6 Q_2 + \frac{\tau ^2 Z_1 Q^2_1 Q^2_4}{K_1 \xi _{{M}}}\left( 1+ \frac{K_2}{K_4} + \frac{K_3}{K_5}+ Q_0\right) \\&\qquad +\frac{\tau Z_2 K_2}{K_4 K_7}\left( \frac{\lambda _{RS} Q_0}{K_6} + 1 + Q_0\right) +\frac{\tau Z_3 K_3}{K_5 K_7}\left( \frac{\lambda _{RS} Q_0}{K_6} + 1 + Q_0\right) \\&\qquad +\frac{\tau Z_7 Q_4 Q_1 K_2}{\xi _{{M}} K_4}+\frac{\tau Z_5 Q_4 Q_1 K_3}{\xi _{{M}} K_5} + \frac{2\tau Z_2 K^2_2}{K^2_4 K_7}+ \frac{2\tau Z_3 K^2_3}{K^2_5 K_7},\\ \eta _2&:= \, \frac{\tau Z_2 K_1 K_2}{K_4 K_6 K_7}+\frac{\tau Z_3 K_1 K_3}{K_5 K_6 K_7}+\frac{\tau Z_4 K_2 K_3}{K_4 K_5 K_7} + Z_6 Q_3.\\ \end{aligned}$$

Since $\left( D_i,\eta _i\right) \in {\mathbb {R}}_{\tiny {>0}} \times {\mathbb {R}}_{\tiny {>0}}$ for $i=1,2$, it follows that the sign of *a* is completely dependent on the size of the quantity $\frac{\eta _2}{\eta _1}$. Therefore, define

$$\begin{aligned} \varLambda :=\frac{\eta _2}{\eta _1} \end{aligned}$$

to arrive at the following dichotomy

$$\begin{aligned} {\left\{ \begin{array}{ll} a<0 \iff \varLambda <1, \\ a>0 \iff \varLambda >1. \\ \end{array}\right. } \end{aligned}$$

This completes the proof concerning the bifurcation analysis of model (4). $\square$

### Summary of Stability Theorems

The goal of this section of the appendix is to provide the reader with a brief collection and overview of the theorems that are widely used in determining the stability of the equilibrium points for nonlinear dynamical systems. The first theorem will be needed in order to determine the local asymptotic stability of the DFE corresponding to the $SEY AR$ model, while the second is used to investigate the existence of non-trivial sub-threshold equilibrium states of the model. In the setting of dynamical systems, one cannot usually pinpoint a solution exactly, but only approximately. As a result, an equilibrium point must be stable to be physically meaningful. A stable equilibrium point of a system is a solution ${\mathbf {x}}^\star$ with the property that if for every open ball $B({\mathbf {x}}^\star ,\epsilon )$ of radius $\epsilon$, centered at ${\mathbf {x}}^\star$, there is a $\delta <\epsilon$, such that if every solution ${\mathbf {x}}$ with initial data ${\mathbf {x}}(0) \in B({\mathbf {x}}^\star ,\delta )$, remains in $B({\mathbf {x}}^\star ,\epsilon )$ for $t>0$. In other words, if the initial data start in $B({\mathbf {x}}^\star ,\delta )$, then the flow map $\phi (t,{\mathbf {x}})$ of the model remains in $B({\mathbf {x}}^\star ,\epsilon )$ for eternity. An equilibrium point ${\mathbf {x}}^\star$ is said to be asymptotically stable, if in addition to the above, there is a $\delta >0$ such that

$$\begin{aligned} \lim _{t \rightarrow +\infty } {\mathbf {x}}(t)={\mathbf {x}}^\star . \end{aligned}$$

Provided an epidemiological model can be grouped into *n* homogeneous compartments, the local asymptotic stability of the equilibrium states can be established by utilizing the next-generation method, appearing in Van den Driessche and Watmough (2002). By making use of the center manifold theorem (John and Philip 1997), Van den Driessche and Watmough provided a simple prescription for determining the local asymptotic stability of DFE points of a given system. This criterion is given in terms of the reproductive number ${\mathcal {R}}_0$ of the system which acts as a threshold value. This effectively relates ${\mathcal {R}}_0$ to the DFE of the system. As a result, this has proven to be very useful in disease control. To cast the above discussion into a mathematical framework, we let ${\mathbf {x}}=\left( x_1,\ldots ,x_k\right) ^T \in {\mathbb {R}}^k_+$ and define the space of disease-free states for the compartmental model to be $X:=\left\{ x \in {\mathbb {R}}^k_+ {:} x_i=0 \text {for} i=1,\cdots ,m, \ m<n\right\}$. Then, for $\varPhi \in C^2({\mathbb {R}}^k)$, we form the following dynamical system:

17 $$\begin{aligned} \dot{{\mathbf {x}}}(t)=\varPhi \left( {\mathbf {x}}(t)\right) ={\mathcal {F}}\left( {\mathbf {x}}(t)\right) -{\mathcal {V}}\left( {\mathbf {x}} (t)\right) , \end{aligned}$$

where $${\mathcal {V}}={\mathcal {V}}^{-}-{\mathcal {V}}^{+}.$$

#### Theorem 6

(Van den Driessche and Watmough 2002) Define ${\mathcal {R}}_0=\rho \left( FV^{-1}\right)$ and consider the disease transmission model given by (17) such that $\varPhi$ satisfies the follow criteria:

i If ${\mathbf {x}} \ge 0$, then so are ${\mathcal {F}}$, ${\mathcal {V}}^{+}$, and ${\mathcal {V}}^{-}$,
ii If ${\mathbf {x}} = 0$, then ${\mathcal {V}}^{-}=0$,
iii If $i>m$, then ${\mathcal {F}}_i=0$,
iv If ${\mathbf {x}} \in X$, then ${\mathcal {F}}_i={\mathcal {V}}^{+}=0$, for all $0 \le i \le m$,
v If ${\mathcal {F}}({\mathbf {x}})=0$, then all eigenvalues of $D\varPhi ({\mathbf {x}}^\star )$ have negative real parts.

If ${\mathbf {x}}^\star$ is a DFE for (17), then ${\mathbf {x}}^\star$ is locally asymptotically stable provided ${\mathcal {R}}_0<1$ and unstable if ${\mathcal {R}}_0>1$.

The above theorem is proved by making use of the following lemma.

#### Lemma 2

If ${\mathbf {x}}^\star$ is a DFE of system (17) and $\varPhi$ satisfies assumptions (i)–(v), then the derivatives $D{\mathcal {F}}({\mathbf {x}}^\star )$ and $D{\mathcal {V}}({\mathbf {x}}^\star )$ can be partitioned as follows:

$$\begin{aligned} D{\mathcal {F}}({\mathbf {x}}^\star )= \begin{pmatrix} F &{}\quad 0 \\ 0 &{}\quad 0 \\ \end{pmatrix} \quad \text {and} \quad D{\mathcal {V}}({\mathbf {x}}^\star )= \begin{pmatrix} V &{}\quad 0 \\ J_3 &{}\quad J_4 \\ \end{pmatrix}, \end{aligned}$$

where *F* and *V* are defined as:

$$\begin{aligned} F= \begin{pmatrix} \frac{ \partial {\mathcal {F}}_i}{\partial x_j} \Big |_{{\mathbf {x}}^\star } \\ \end{pmatrix}_{1 \le i,j \le m} \quad \text {and} \quad V= \begin{pmatrix} \frac{ \partial {\mathcal {V}}_i}{\partial x_j} \Big |_{{\mathbf {x}}^\star } \\ \end{pmatrix}_{1 \le i,j \le m}. \end{aligned}$$

In the above matrix partitioning, *F* is non-negative, *V* is an invertible *M*-matrix, and $J_3$, $J_4$ are sub-matrices of the Jacobian associated with various transmission terms. This theorem provides a convenient epidemiological interpretation of the reproductive threshold ${\mathcal {R}}_0$ corresponding to a given dynamical system in the SIR family. Additionally, due to the partitioning of the Jacobian mentioned above, the stability of the system is determined by $\det {\left( FV^{-1}-\lambda {\mathcal {I}} \right) }$, where ${\mathcal {I}}$ is the identity matrix. If the matrix *F* containing transmission probabilities and contact rates is set to zero, then all eigenvalues of $-V$ have negative real part. As a result, the stability, or lack of, experienced by the system in question depends on the entries of *F*. Due to this reason, the bifurcation parameters are chosen from the entries of *F*. In the case that the transmission probabilities are relatively high, an endemic could occur and additional non-trivial equilibrium can arise. Regarding the $SEY AR$ model, besides the human biting rate $\sigma$, the only possible parameters are the transmission probabilities $\beta _Y$, $\beta _A$ and $\beta _{{M}}$. From a epidemiological perspective, we have more control over the mosquito-to-human transmission probability $\beta _{{M}}$. Due to this reason, $\beta _{{M}}$ is chosen as the parameter for the bifurcation analysis. One could perform a similar analysis for $\beta _Y$ and $\beta _A$; however, all of the transmission probabilities involved are related through the reproductive number ${\mathcal {R}}_0$ of the model. As a result, the analysis would be similar.

Let *s*(*A*) and $\rho (A)$ stand for the spectral abscissa and radius, respectively. The proof of Theorem 6 hinges on M-matrix theory. A matrix *B* is said to have the *Z*-sign pattern, provided all of its off diagonal entries are non-positive. If $B=s {\mathcal {I}}-P$, where $P \ge 0$. If $s>\rho (P)$, then *B* is a non-singular M-matrix; if $s=\rho (P)$, then *B* is a singular M-matrix. This observation is then combined with a linear algebra lemma, which we restate below for convenience of the reader. To this end, they make use of the following argument: define $J_1:=F-V$, then *V* is a non-singular M-matrix and *F* is non-negative. It follows that $-J_1:=V-F$ has the *Z*-sign pattern. By the non-negativity of $FV^{-1}$, it is a direct consequence that $-J_1V^{-1}:={\mathcal {I}}-FV^{-1}$ also has the *Z*-sign pattern. Now we apply Lemma 3 with $H=V$ and $-J_1:=V-F$, to conclude that $-J_1$ is a non-singular M-matrix if and only if ${\mathcal {I}}-FV^{-1}$. Also, since all of its eigenvalues have magnitude bounded above by $\rho (FV^{-1})$, we have that ${\mathcal {I}}-FV^{-1}$ is a non-singular M-matrix if and only if $\rho (FV^{-1})<1$. Therefore, $s(J_1)<0$ if and only if ${\mathcal {R}}_0<1$. Through making use of a similar argument in combination with Lemma 6 of Appendix A, found in Van den Driessche and Watmough (2002), they arrive at the following trichotomy, establishing ${\mathcal {R}}_0$ as a threshold parameter

$$\begin{aligned} {\left\{ \begin{array}{ll} s(J_1)<0 \iff {\mathcal {R}}_0<1,\\ s(J_1)=0 \iff {\mathcal {R}}_0=1,\\ s(J_1)>0 \iff {\mathcal {R}}_0>1.\\ \end{array}\right. } \end{aligned}$$

#### Lemma 3

Let *H* be a non-singular M-matrix and assume that *B* and $BH^{-1}$ have the *Z*-sign pattern. Then, *B* is a non-singular M-matrix if and only if $BH^{-1}$ is a non-singular M-matrix.

In Sect. 5 use is made of the following variant of the center manifold theorem specifically adapted to the case of bifurcation analysis for nonlinear systems. The utility of this theorem resides in the classification of the bifurcation due to the sign of the parameters *a* and *b*, defined below.

#### Theorem 7

(Castillo-Chavez and Song 2004) Consider the following dynamical system with real parameter $\omega$:

$$\begin{aligned} {\dot{x}}=f(x,\omega ),\quad f:{\mathbb {R}}^n \times {\mathbb {R}} \rightarrow {\mathbb {R}},\quad \text {and} \quad f \in C^2({\mathbb {R}}^n \times {\mathbb {R}}). \end{aligned}$$

Without loss of generality, we assume that $x=0$ is an equilibrium point for the above system for all $\omega$, i.e., $f(0,\omega ) \equiv 0$ for all $\omega$. Provided that the following assumptions are satisfied:

I $A=D_x f(0,0)=\Big ( \frac{\partial f_i}{\partial x_j} (0,0)\Big )$ is the linearization matrix of the system around the equilibrium point 0 with $\omega$ evaluated at 0. Zero is a simple eigenvalue of *A*, and all remaining eigenvalues have negative real part. Non-trivial null space of dimension one.
II The matrix *A* has a right eigenvector *w* and left eigenvector *v* corresponding to the zero eigenvalue.

Let $f_k$ denote the $k\text {th}$ component of *f* and

18 $$\begin{aligned} a&= \sum _{i,j,k=1}^{n} v_k w_i w_j \frac{\partial ^2 f_k}{\partial x_i \partial x_j}\left( 0,0\right) , \end{aligned}$$

19 $$\begin{aligned} b&=\sum _{i,k=1}^{n} v_k w_i \frac{\partial ^2 f_k}{\partial x_i \partial \omega }\left( 0,0\right) . \end{aligned}$$

Then, the local dynamics around the equilibrium point 0 are completely determined by the signs of *a* and *b*. More precisely,

i $a>0$, $b>0$. When $\omega <0$ such that $|\omega | \ll 1$, 0 is locally asymptotically stable and there exists a positive unstable equilibrium; when $0<\omega \ll 1$, 0 is unstable and there exists a negative locally asymptotically stable equilibrium.
ii $a<0$, $b<0$. When $\omega <0$ such that $|\omega | \ll 1$, 0 is unstable; when $0<\omega \ll 1$, 0 is locally asymptotically stable and there exists a positive unstable equilibrium.
iii $a>0$, $b<0$. When $\omega <0$ such that $|\omega | \ll 1$, 0 is unstable and there exists a locally asymptotically stable negative equilibrium; when $0<\omega \ll 1$, 0 is stable and a positive unstable equilibrium exists.
iv $a<0$, $b>0$. When $\omega$ changes sign, 0 changes its stability from stable to unstable. As a result, a negative unstable equilibrium becomes positive and locally asymptotically stable. In particular, if $a>0$ and $b>0$, then a backward bifurcation occurs at $\omega =0$.

The *a* and *b* terms appearing in the above theorem depend on generalized eigenvectors, i.e., zero entries are allowed. In the proof of Theorem 7, the *a* and *b* terms arise from a differential equation obtained from a parameterization of a one-dimensional center manifold *c*(*t*) given by

$$\begin{aligned} {\dot{c}}=\frac{a}{2}c^2+b\omega c. \end{aligned}$$

Observe how a transcritical bifurcation occurs in the above equation at $\omega =0$ and can be classified according to the signs of the *a* and *b* terms, defined above. These terms depend on the Kronecker product of generalized eigenvectors and entries of the Hessian evaluated at the DFE. As pointed out by Castillo-Chavez and Song (2004), negative components of the generalized eigenvectors are permitted through a modification of Theorem 7. The essence of the argument hinges on the fact that the theorem can still be applied; one only has to compare the negative entries of the eigenvectors with their corresponding entries of the nonnegative equilibrium of interest. Therefore, one has to consider the original parameterization of the center manifold, prior to the coordinate change where the DFE is assumed to be zero. Notice how the negative entries in the generalized eigenvectors calculated in Sect. 5 correspond to the positive entries of the DFE of interest.

### Parameter Values

Presented below are tables of numerical rates corresponding to the following three high transmission sites: Kaduna in Nigeria, Namawala in Tanzania, and Butelgut in Papua New Guinea. The data have been collected from multiple sources, all of which are noted in the footnotes. Due to the additional prevalence of *P. vivax* in Butelgut, the corresponding data represent combined estimates of both species (Killeen et al. 2000). The data for the other sites exclusively correspond to the *P. falciparum* species. There are a variety of vector species that inhabit these sites. The data listed here correspond to the dominant vector species of the area being considered. The dominant vector species of each site is listed in the footnotes. Furthermore, as mentioned in Sect. 2 and reported by Laishram et al. (2012a), the parasites carried by asymptomatic human hosts can be more infectious than those of symptomatic. For the parameter values displayed in “Parameter Values” of appendix, we invoke the assumption that the asymptomatic carriers corresponding to each site transmit at a lower rate than that of the symptomatic and that asymptomatic individuals recover faster than symptomatic, i.e., $\beta _A<\beta _Y$ and $\lambda _{AR}>\lambda _{YR}$.

Table 4 contains demographic data for the countries that each site is contained in. These numerical values are used to calculate the recruitment rates of the human populations $\varOmega _{{H}}$ corresponding to each region under consideration (Tables 5, 6, 7).

**Table 4:** Human population data

Country	Life expectancy	Birth rate	Migration rate	Source
Nigeria	53.02	37.64	$-$ 0.22	Central Intelligence Agency (2015)
Tanzania	61.71	36.39	$-$ 0.54	Central Intelligence Agency (2015)
Papua New Guinea	67.03	24.38	0.00	Central Intelligence Agency (2015)

The human population data displayed in Table 4 are from the Central Intelligence Agency (CIA). $\varOmega _{_{{{\textit{LE}}}}}$, $\varOmega _{_{{{\textit{BR}}}}}$, and $\varOmega _{_{{{\textit{MR}}}}}$ denote the life expectancy, birth rate, and migration rate of the population under consideration. Life expectancy is measured in years. The birth rate entries appearing in column 3 of the above table are crude birth rates. The crude birth rate measures the average quantity of live births during a year, per 1000 people, and is given in units of total births per 1000 people per year. The migration rates are net migration rates, which measure the difference of immigrants and emigrants in a given population over the span of a year, and are given in units of humans per year, per 1000 people

**Table 5:** Kaduna

Parameter	Dimension	Value	Source
$$\varOmega _{{H}}$$	Humans $$\times $$$$\text {days}^{-1}$$	$$1.02 \times 10^{-4}$$	Central Intelligence Agency (2015)$${}^\mathrm{a}$$
$$\varOmega _{{M}}$$	Mosquitoes $$\times $$$$\text {days}^{-1}$$	1505.82	Killeen et al. (2000)$${}^\mathrm{b}$$
$\xi _{{H}}$	$\text {days}^{-1}$	0.019	Central Intelligence Agency (2015)${}^\mathrm{c}$
$\xi _{{M}}$	$\text {days}^{-1}$	0.11	Molineaux et al. (1979) and Chitnis et al. (2008)${}^\mathrm{d}$
$\beta _A$	n/a	0.048	Assumed${}^\mathrm{e}$
$\beta _Y$	n/a	0.48	Chitnis et al. (2008)${}^\mathrm{f}$
$\beta _{{M}}$	n/a	0.032	Killeen et al. (2000)${}^\mathrm{g}$
$\gamma$	$\text {days}^{-1}$	0.11	Chitnis et al. (2008)${}^\mathrm{h}$
$\tau$	$\text {days}^{-1}$	0.09	Killeen et al. (2000) and Craig et al. (1999)${}^\mathrm{i}$
$\delta$	$\text {days}^{-1}$	$1.66 \times 10^{-6}$	World Life Expectancy (2016)${}^\mathrm{j}$
$\sigma$	$\text {days}^{-1}$	0.42	Killeen et al. (2000)${}^\mathrm{k}$
$\lambda _{AR}$	$\text {days}^{-1}$	0.6	Assumed${}^\mathrm{l}$
$\lambda _{YR}$	$\text {days}^{-1}$	0.06	Assumed${}^\mathrm{m}$
$\lambda _{RS}$	$\text {days}^{-1}$	$5.48 \times 10^{-4}$	Chitnis et al. (2008)${}^\mathrm{n}$
$u_{_{{\textit{low}}}}$	n/a	0.5	Assumed${}^\mathrm{o}$

$^{\mathrm{a}}$ The human recruitment rate is obtained from the entries in the first row of Table 4 and the weighted sum formula $\varOmega _{{H}}=(\varOmega _{_{{{\textit{BR}}}}} +\varOmega _{_{{{\textit{MR}}}}})/365.25/1000=(37.64-0.22)/365.25/1000 \approx 1.02 \times 10^{-4}$

$^\mathrm{b}$ The daily vector emergence rate is measured in units of new adult female mosquitoes per day. Thus, it is given by dividing the entry in row six column four of Table 3 in Killeen et al. (2000, p. 541) by the normalizing quantity 365.25, i.e., $\varOmega _{{M}}=(0.55 \times 10^{6})/365.25 \approx 1505.82$

$^{\mathrm{c}}$ The average natural human mortality rate is calculated by dividing the entry listed in row one column two of Table 4 into unity, i.e., $\xi _{{H}}=1/\varOmega _{_{{{\textit{LE}}}}} =1/53.02 \approx 0.019$

$^{\mathrm{d}}$ The dominant vector species of Kaduna at the time the field measurements were taken was *An. gambiae* (Service 1965). Let $\varOmega _{_{{{\textit{L}}}}}$ stand for the average *An. gambiae* life expectancy, which is dependent upon the region under consideration. The data provided by Molineaux et al. (1979) and appearing in row nine column one of Table A.3 in Chitnis et al. (2008, p. 19) correspond to *An. gambiae* activity in Nigeria. Due to this, we select the entry contained in row one column nine, so that $\varOmega _{_{{{\textit{L}}}}}=9$. Therefore, the average daily *An. gambiae* mosquito mortality rate is $\xi _{{M}}=1/\varOmega _{_{{{\textit{L}}}}}=1/9 \approx 0.11$

$^{\mathrm{e}}$ The assumption is made that the rate of transmission from asymptomatic humans to susceptible mosquitoes is one-tenth the transmission probability corresponding to symptomatic humans, i.e., $\beta _A=0.048$

$^{\mathrm{f}}$ We adopt the convention utilized by Chitnis et al. (2008), where an estimate of 0.48 will be used for high transmission areas and an estimate of 0.24 will be used for low. Kaduna is a high transmission area; thus, we let $\beta _Y=0.48$. An alternate choice would be to choose the average value of the parameters corresponding to the dominant species of parasite. The average value of the parameters appearing in rows one through five in column one of Table A.6 in Chitnis et al. (2008, p. 21) is approximately 0.36. The origin of the parameters used in this calculation is Thomson et al. (1957), Boyd (1949) and Draper (1953)

$^{\mathrm{g}}$ The effective daily vector-to-human contact rate is obtained by dividing the value in row two column four of Table 3 in Killeen et al. (2000, p. 541) by $\varOmega _{_{{{\textit{L}}}}}$. Thus, $\beta _{{M}}=0.29 / \varOmega _{_{{{\textit{L}}}}}=0.29 / 9 \approx 0.032$

$^\mathrm{h}$ The human latent period ${\tilde{\gamma }}$, measure in days, corresponding to *P. falciparum* infection is taken from row three column one in Table A.7 in Chitnis et al. (2008, p. 21). The range 9–10 is given. The average value is then chosen, i.e., ${\tilde{\gamma }}\approx 9.5$. It follows that the average duration of the intermediate host latent period is $\gamma =1 / {\tilde{\gamma }}=1 / 9.5 \approx 0.11$. The parameter source is Molineaux and Gramiccia (1980)

$^\mathrm{i}$ Let ${\tilde{\tau }}$ denote the *Plasmodium* incubation period, i.e., the number of days required for parasite development. Thus, by using the entry in row nine column three of Table 2 in Killeen et al. (2000, p. 539), it follows that the average duration of the definitive host latent period is $\tau =1 / {\tilde{\tau }}=1 / 11.6 \approx 0.09$. Technically, this parameter was calculated from the mean and median temperatures listed in the original source (Craig et al. 1999)

$^\mathrm{j}$ The malaria death rates are taken from World Life Expectancy (2016) and are given in units of per 100, 000 people per year. As in the case of the human demographic data listed in Table 4, these rates correspond to the overall country that the region is contained in. Using the data provided by World Life Expectancy (2016), it follows that $\delta =60.46/365.25/100,000 \approx 1.66 \times 10^{-6}$

$^\mathrm{k}$ The average human biting rate is estimated by dividing the entry in row one column four of Table 3 in Killeen et al. (2000, p. 541) by $\varOmega _{_{{{\textit{L}}}}}$. More precisely, we have that $\sigma =3.8/9 \approx 0.42$

$^\mathrm{l,m}$ For the purpose of numerical simulations, we assume that asymptomatic carriers transmit malaria to a lesser extent than symptomatic carriers and recover faster. Due to this, we assume that the recovery rate $\lambda _{YR}$ for symptomatic individuals is one-tenth the rate $\lambda _{AR}$ of asymptomatic individuals. Let the quantity $1/\lambda$ denote the average duration of the human infectious period, provided that the individual has had no treatment. If $\varOmega _{{I}}$ stands for the average duration of the *P. falciparum* infectious period in humans, we select the average of the entries appearing in row four column one of Table A.9 in Chitnis et al. (2008, p. 21), so that $\varOmega _{{I}}=18$. Therefore, $\lambda _{YR} \approx \lambda =1/ \varOmega _{{I}}=1 /18 \approx 0.06$, and it follows that $\lambda _{AR}=0.6$. The original source of the parameter value is Bloland and Williams (2002)

$^\mathrm{n}$ The temporary immunity loss rate $\lambda _{RS}$ is such that $1 / \lambda _{RS}$ is equal to the average duration of the human immune period. As in Chitnis et al. (2008), we assume that the immune period lasts for an average of 5 years in areas of high transmission and 1 year in areas of low transmission. For Kaduna, it follows that $\lambda _{RS}=1 / (5)365.25 \approx 5.48 \times 10^{-4}$

$^\mathrm{o}$ This baseline assumption is due to the fact that the population under consideration is $A{\textit{-dominant}}$

**Table 6:** Namawala

Parameter	Dimension	Value	Source
$$\varOmega _{{H}}$$	humans $$\times $$$$\text {days}^{-1}$$	$$9.82 \times 10^{-5}$$	Central Intelligence Agency (2015)$${}^\mathrm{a}$$
$$\varOmega _{{M}}$$	mosquitoes $$\times $$$$\text {days}^{-1}$$	4928.13	Killeen et al. (2000)$${}^\mathrm{b}$$
$\xi _{{H}}$	$\text {days}^{-1}$	0.016	Central Intelligence Agency (2015)${}^\mathrm{c}$
$\xi _{{M}}$	$\text {days}^{-1}$	0.09	Molineaux et al. (1979) and Chitnis et al. (2008)${}^\mathrm{d}$
$\beta _A$	n/a	0.048	Assumed${}^\mathrm{e}$
$\beta _Y$	n/a	0.48	Chitnis et al. (2008)${}^\mathrm{f}$
$\beta _{{M}}$	n/a	0.002	Killeen et al. (2000)${}^\mathrm{g}$
$\gamma$	$\text {days}^{-1}$	0.11	Chitnis et al. (2008)${}^\mathrm{h}$
$\tau$	$\text {days}^{-1}$	0.09	Killeen et al. (2000) and Craig et al. (1999)${}^\mathrm{i}$
$\delta$	$\text {days}^{-1}$	$1.16 \times 10^{-6}$	World Life Expectancy (2016)${}^\mathrm{j}$
$\sigma$	$\text {days}^{-1}$	0.13	Killeen et al. (2000)${}^\mathrm{k}$
$\lambda _{AR}$	$\text {days}^{-1}$	0.6	Assumed${}^\mathrm{l}$
$\lambda _{YR}$	$\text {days}^{-1}$	0.06	Assumed${}^\mathrm{m}$
$\lambda _{RS}$	$\text {days}^{-1}$	$5.48 \times 10^{-4}$	Chitnis et al. (2008)${}^\mathrm{n}$
$u_{_{{\textit{low}}}}$	n/a	0.5	Assumed${}^\mathrm{o}$

$^\mathrm{a}$ From Table 4, it follows that $\varOmega _{{H}}=(36.39-0.54)/365.25/1000 \approx 9.82 \times 10^{-5}$

$^\mathrm{b}$ By utilizing the data provided in Table 3 in Killeen et al. (2000, p. 541), it follows that $\varOmega _{{M}}=(1.8 \times 10^{6})/365.25 \approx 4928.13$

$^\mathrm{c}$ The average natural human mortality rate is calculated in a similar fashion, as in the case of Kaduna, by utilizing the data provided in Table 4

$^\mathrm{d}$ The dominant vector species of Namawala at the time the field measurements were taken was *An. gambiae* (Smith et al. 1993). The data provided by Gillies and Wilkes (1965) and appearing in row six column one of Table A.3 in Chitnis et al. (2008, p. 19) correspond to *An. gambiae* activity in Tanzania. Using these data, it follows that $\varOmega _{_{{{\textit{L}}}}}=11.26$. Therefore, the average daily *An. gambiae* mosquito mortality rate is $\xi _{{M}}=1/11.26 \approx 0.09$

$^\mathrm{e}$ As previously mentioned, the asymptomatic human-to-susceptible mosquito transmission probability is assumed to be $\beta _A=0.048$

$^\mathrm{f}$ Since Namawala is a high transmission area, we let $\beta _Y=0.48$

$^\mathrm{g}$ By dividing the value in row two column five of Table 3 in Killeen et al. (2000, p. 541) by $\varOmega _{_{{{\textit{L}}}}}$, it follows that $\beta _{{M}}=0.017 / 11.26 \approx 0.002$

$^\mathrm{h}$ The average duration of the intermediate host latent period is taken to be $\gamma =1 / {\tilde{\gamma }}=1 / 9.5 \approx 0.11$. The parameter value ${\tilde{\gamma }}$ is taken from row three column one in Table A.7 in Chitnis et al. (2008, p. 21). The average value is then chosen, i.e., $\gamma \approx 9.5$. The original parameter source is Molineaux and Gramiccia (1980)

$^\mathrm{i}$ By using the entry in row eight column four of Table 2 in Killeen et al. (2000, p. 539), it follows that the average duration of the definitive host latent period is $\tau =1 / 11.6 \approx 0.09$

$^\mathrm{j}$ By the data provided by World Life Expectancy (2016), it follows that $\delta =42.42/365.25/100,000 \approx 1.16 \times 10^{-6}$

$^\mathrm{k}$ By using the data in row one column five of Table 3 in Killeen et al. (2000, p. 541), we have that $\sigma =1.5/11.26 \approx 0.13$

$^\mathrm{l}$ As in the previous table, the recovery rate pertaining to asymptomatic individuals is assumed to be $\lambda _{AR}=0.6$

$^\mathrm{m}$ The average of the entries appearing in row four column one of Table A.9 in Chitnis et al. (2008, p. 21) is selected, so that $\varOmega _{{I}}=18$. Therefore, $\lambda _{YR} \approx \lambda =1/ \varOmega _{{I}}=1 /18 \approx 0.06$. The original source of the parameter value is Bloland and Williams (2002)

$^\mathrm{n}$ As Namawala is a high transmission area, the temporary immunity loss rate is taken to be $\lambda _{RS}=1 / (5)365.25 \approx 5.48 \times 10^{-4}$

$^\mathrm{o}$ This baseline assumption is due to the fact that the population under consideration is $A{\textit{-dominant}}$

**Table 7:** Butelgut

Parameter	Dimension	Value	Source
$$\varOmega _{{H}}$$	humans $$\times $$$$\text {days}^{-1}$$	$$6.67 \times 10^{-5}$$	Central Intelligence Agency (2015)$${}^\mathrm{a}$$
$$\varOmega _{{M}}$$	mosquitoes $$\times $$$$\text {days}^{-1}$$	4106.78	Killeen et al. (2000)$${}^\mathrm{b}$$
$\xi _{{H}}$	$\text {days}^{-1}$	0.015	Central Intelligence Agency (2015)${}^\mathrm{c}$
$\xi _{{M}}$	$\text {days}^{-1}$	0.14	Molineaux et al. (1979) and Chitnis et al. (2008)${}^\mathrm{d}$
$\beta _A$	n/a	0.048	Assumed${}^\mathrm{e}$
$\beta _Y$	n/a	0.48	Chitnis et al. (2008)${}^\mathrm{f}$
$\beta _{{M}}$	n/a	0.006	Killeen et al. (2000)${}^\mathrm{g}$
$\gamma$	$\text {days}^{-1}$	0.11	Chitnis et al. (2008)${}^\mathrm{h}$
$\tau$	$\text {days}^{-1}$	0.1	Killeen et al. (2000) and Craig et al. (1999)${}^\mathrm{i}$
$\delta$	$\text {days}^{-1}$	$1.03 \times 10^{-6}$	World Life Expectancy (2016)${}^\mathrm{j}$
$\sigma$	$\text {days}^{-1}$	0.14	Killeen et al. (2000)${}^\mathrm{k}$
$\lambda _{AR}$	$\text {days}^{-1}$	0.6	Assumed${}^\mathrm{l}$
$\lambda _{YR}$	$\text {days}^{-1}$	0.06	Assumed${}^\mathrm{m}$
$\lambda _{RS}$	$\text {days}^{-1}$	$5.48 \times 10^{-4}$	Chitnis et al. (2008)${}^\mathrm{n}$
$u_{_{{\textit{low}}}}$	n/a	0.5	Assumed${}^\mathrm{o}$

$^\mathrm{a}$ From Table 4, it follows that $\varOmega _{{H}}=(24.38-0.00)/365.25/1000 \approx 6.67 \times 10^{-5}$

$^\mathrm{b}$ By utilizing the data provided in Table 3 in Killeen et al. (2000, p. 541), it follows that $\varOmega _{{M}}=(1.5 \times 10^{6})/365.25\approx 4106.78$

$^\mathrm{c}$ Similarly, the average natural human mortality rate is calculated by utilizing the data provided in Table 4

$^\mathrm{d}$ The dominant vector species of Butelgut at the time the field measurements were taken was *An. punctulatus* (Burkot et al. 1988). The data provided by Peters and Standfast (1960) and appearing in row thirteen column one of Table A.3 in Chitnis et al. (2008, p. 19) correspond to *An. punctulatus* activity in Papua New Guinea. Using these data, it follows that $\varOmega _{_{{{\textit{L}}}}}=7.1$. Therefore, the average daily *An. punctulatus* mosquito mortality rate is $\xi _{{M}}=1/7.1 \approx 0.14$

$^\mathrm{e}$ As previously demonstrated, the asymptomatic human-to-susceptible mosquito transmission probability is assumed to be $\beta _A=0.048$

$^\mathrm{f}$ Since Butelgut is a high transmission area, we let $\beta _Y=0.48$

$^\mathrm{g}$ By dividing the value in row two column six of Table 3 in Killeen et al. (2000, p. 541) by $\varOmega _{_{{{\textit{L}}}}}$, it follows that $\beta _{{M}}=0.042 / 7.1 \approx 0.006$

$^\mathrm{h}$ The average duration of the intermediate host latent period is taken to be $\gamma =1 / {\tilde{\gamma }}=1 / 9.5 \approx 0.11$. The parameter value ${\tilde{\gamma }}$ is taken from row three column one in Table A.7 in Chitnis et al. (2008, p. 21). The average value is then chosen, i.e., $\gamma \approx 9.5$. The original parameter source is Molineaux and Gramiccia (1980)

$^\mathrm{i}$ By using the entry in row eight column five of Table 2 in Killeen et al. (2000, p. 539), it follows that the average duration of the definitive host latent period is $\tau =1 / 9.6 \approx 0.1$

$^\mathrm{j}$ By the data provided by World Life Expectancy (2016), it follows that $\delta =37.57/365.25/100{,}000 \approx 1.03 \times 10^{-6}$

$^\mathrm{k}$ By using the data in row one column six of Table 3 in Killeen et al. (2000, p. 541), we have that $\sigma =0.99/\varOmega _{_{{{\textit{L}}}}}=0.99/7.1 \approx 0.14$

$^\mathrm{l}$ Due to similar reasoning as above, the recovery rate pertaining to asymptomatic individuals is assumed to be $\lambda _{AR}=0.6$

$^\mathrm{m}$ The average of the entries appearing in row four column one of Table A.9 in Chitnis et al. (2008, p. 21) is selected, so that $\varOmega _{{I}}=18$. Therefore, $\lambda _{YR} \approx \lambda =1/ \varOmega _{{I}}=1 /18 \approx 0.06$. The original source of the parameter value is Bloland and Williams (2002)

$^\mathrm{n}$ As Butelgut is a high transmission area, the temporary immunity loss rate is taken to be $\lambda _{RS}=1 / (5)365.25 \approx 5.48 \times 10^{-4}$

$\mathrm{o}$ This baseline assumption is due to the fact that the population under consideration is $A{\textit{-dominant}}$
